# Targeting the ANKRD54/ELOVL6 axis suppresses malignancy in BRAF mutant colorectal cancer by disrupting fatty acid metabolism and promoting ferroptosis

**DOI:** 10.1186/s13046-026-03809-0

**Published:** 2026-08-11

**Authors:** Yichao Hou, Di Wu, Yanyu Wei, Bohui Yu, Donghan Shao, Dachun Cao, Ping Wang, Mei Guo, Hang Yin, Li Huo, Wenjun Gu, Yaping Liu, Qingwei Zhang, Dong Wang

**Affiliations:** 1 Shanghai Key Laboratory of Gut Microecology and Associated Major Diseases Research, Digestive Disease Research and Clinical Translation Center, Department of Gastroenterology, Shanghai Ninth People’s Hospital, School of Medicine, Shanghai Jiao Tong University, No. 639 Zhizaoju Road, Shanghai, China; 2 Department of Gastroenterology, Shanghai Ninth People’s Hospital, School of Medicine, Shanghai Jiao Tong University, Shanghai, China; 3 Division of Gastroenterology and Hepatology, Key Laboratory Gastroenterology and Hepatology, Ministry of Health, State Key Laboratory for Oncogenes and Related Genes, Renji Hospital, School of Medicine, Shanghai Jiao Tong University, Shanghai Institute of Digestive Disease, No. 160 Pujian Road, Pudong New Area, Shanghai, China; 4 Department of Oncology, East Hospital Affiliated to Tongji University, Tongji University School of Medicine, Tongji University, Shanghai, China

**Keywords:** ANKRD54, BRAF mutation, Colorectal Cancer, ELOVL6, Fatty Acid, Ferroptosis

## Abstract

**Background:**

BRAF-mutant (BRAF-MT) colorectal cancer (CRC) represents a clinically aggressive subtype characterized by distinct biological features and significantly worse prognosis compared to BRAF wild-type (BRAF-WT) CRC, with median survival reduced by approximately 40%. The therapeutic resistance of BRAF-MT CRC stems primarily from BRAF-driven dysregulation of tumor cell proliferation. However, the molecular mechanisms underlying the aggressive phenotype and potential therapeutic vulnerabilities remain incompletely understood.

**Methods:**

Bioinformatic analyses were employed to identify candidate genes. A combination of in vitro (cell proliferation, clonal formation), ex vivo (patient-derived and mouse intestinal organoids), and in vivo (xenograft models) assays were used to assess oncogenic function. Mechanistic investigations included Western blotting, qRT-PCR, co-immunoprecipitation (Co-IP), chromatin immunoprecipitation (ChIP), and luciferase reporter assays to delineate the regulatory axis. Fatty acid metabolism and ferroptosis were evaluated by a combination of metabolomic, biochemical, and cellular assays.

**Results:**

Bioinformatic screening identified ankyrin repeat domain 54 (ANKRD54) as selectively overexpressed in BRAF-MT CRC and significantly associated with adverse prognosis. Inhibition of ANKRD54 markedly suppressed clonogenic potential, cellular proliferation in vitro, organoid formation in both PDOs and cKI mouse-derived organoids, and tumor growth in vivo. Mechanistically, lysine acetyltransferase 2 A (KAT2A) promoted ANKRD54 transcriptional activation through H3K27 acetylation. ANKRD54 subsequently formed a functional complex with CCAAT/enhancer binding protein beta (CEBPB), cooperatively inducing ELOVL fatty acid elongase 6 (ELOVL6) expression. This KAT2A-mediated ANKRD54/CEBPB/ELOVL6 transcriptional axis reprogrammed fatty acid metabolism, selectively suppressing ferroptosis in BRAF-MT CRC. Pharmacological targeting of either ANKRD54 or ELOVL6 substantially enhanced the therapeutic efficacy of Vemurafenib-based treatment in BRAF-MT CRC models. Clinical data corroborated that elevated ANKRD54 expression promoted malignant progression and predicted poor outcomes through this ELOVL6-dependent metabolic pathway.

**Conclusions:**

This study elucidates a novel KAT2A-mediated ANKRD54/CEBPB/ELOVL6 axis driving aggressive tumor behavior through regulating fatty acid metabolic and ferroptosis evasion. These findings establish ANKRD54 and ELOVL6 as promising therapeutic targets for combination strategies to overcome therapeutic resistance in BRAF-MT CRC patients.

**Supplementary Information:**

The online version contains supplementary material available at https://doi.org/10.1186/s13046-026-03809-0.

## Introduction

BRAF mutations (MTs), particularly the BRAF^V600E^ variant, are prevalent oncogenic drivers in colorectal cancer (CRC), malignant melanoma, and thyroid carcinoma [1–3]. These mutations induce constitutive activation of downstream mitogen-activated protein kinase (MAPK) signaling, promoting tumorigenesis through enhanced cell cycle progression and suppression of apoptosis [4]. While the clinical introduction of BRAF inhibitors (BRAFi) and subsequent combination therapy with MEK inhibitors (MEKi) have improved outcomes for some patients with BRAF-MT tumors, a substantial proportion exhibit primary resistance to these targeted therapies [5]. In CRC, BRAF-MT defines a distinct molecular subtype with particularly poor prognosis, accounting for approximately 8–12% of all CRC cases [6]. This subtype frequently co-occurs with microsatellite instability (MSI) and mismatch repair deficiency (dMMR) [1], and is characterized by aggressive clinical behavior and inherent resistance to conventional chemotherapy or targeted therapy [7, 8].

BRAF-MT CRC may reshape its cellular fate by remodeling its own lipid metabolism. Research shows that tumor cells resistant to BRAF-targeted therapy exhibit a high dependence on lipid metabolism [9]. Mutant BRAF also drives abnormal fatty acid (FA) synthesis by reprogramming metabolism and activating sterol-regulatory element binding proteins (SREBP), thereby meeting their demands for cell membrane construction and energy [10]. Since lipid peroxidation serves as a driving force for ferroptosis, this rewired lipid metabolism promotes both proliferation and a ferroptosis-resistant phenotype through dysregulation of lipid peroxidation [11]. Ferroptosis is an iron-dependent form of cell death characterized by lipid peroxidation. Prostaglandin-endoperoxide synthase 2 (PTGS2), a pro-inflammatory enzyme, is typically upregulated during ferroptosis and serves as an oxidative stress biomarker [12], whereas glutathione peroxidase 4 (GPX4) acts as a central regulator of ferroptosis by reducing lipid hydroperoxides [13]. However, the specific molecular mechanisms underlying alterations in lipid metabolism and ferroptosis in BRAF-MT CRC remain unclear.

Ankyrin repeat domain-containing protein 54 (ANKRD54) is a protein whose functions remain unclear. This typical ankyrin repeat structure suggests a potential role as scaffolds in protein-protein interaction network [14]. In small cell cervical cancer, ANKRD has been identified as a high-frequency human papillomavirus (HPV) integration hotspot [15]. Meanwhile, ANKRD29 has been reported to potentially function as a tumor suppressor in non-small cell lung cancer [16]. Nevertheless, the function and role of ANKRD54 in tumors remain elusive.

Here, we identified ANKRD54 overexpression in BRAF-MT CRC, and its targeted inhibition suppressed malignant progression. Mechanistically, lysine acetyltransferase 2 A (KAT2A)-regulated ANKRD54 forms a complex with CCAAT/enhancer binding protein beta (CEBPB) to activate ELOVL fatty acid elongase 6 (ELOVL6), thereby regulating FA metabolism and ferroptosis. Combining ANKRD54 or ELOVL6 inhibition with Vemurafenib enhanced therapeutic efficacy. Thus, our study revealed the ANKRD54/ELOVL6 axis as a metabolic vulnerability for targeted BRAF-MT CRC therapy.

## Results

### ANKRD54 serves as a prognostic indicator predicting poor outcomes in BRAF-MT CRC

**Fig. 1:**
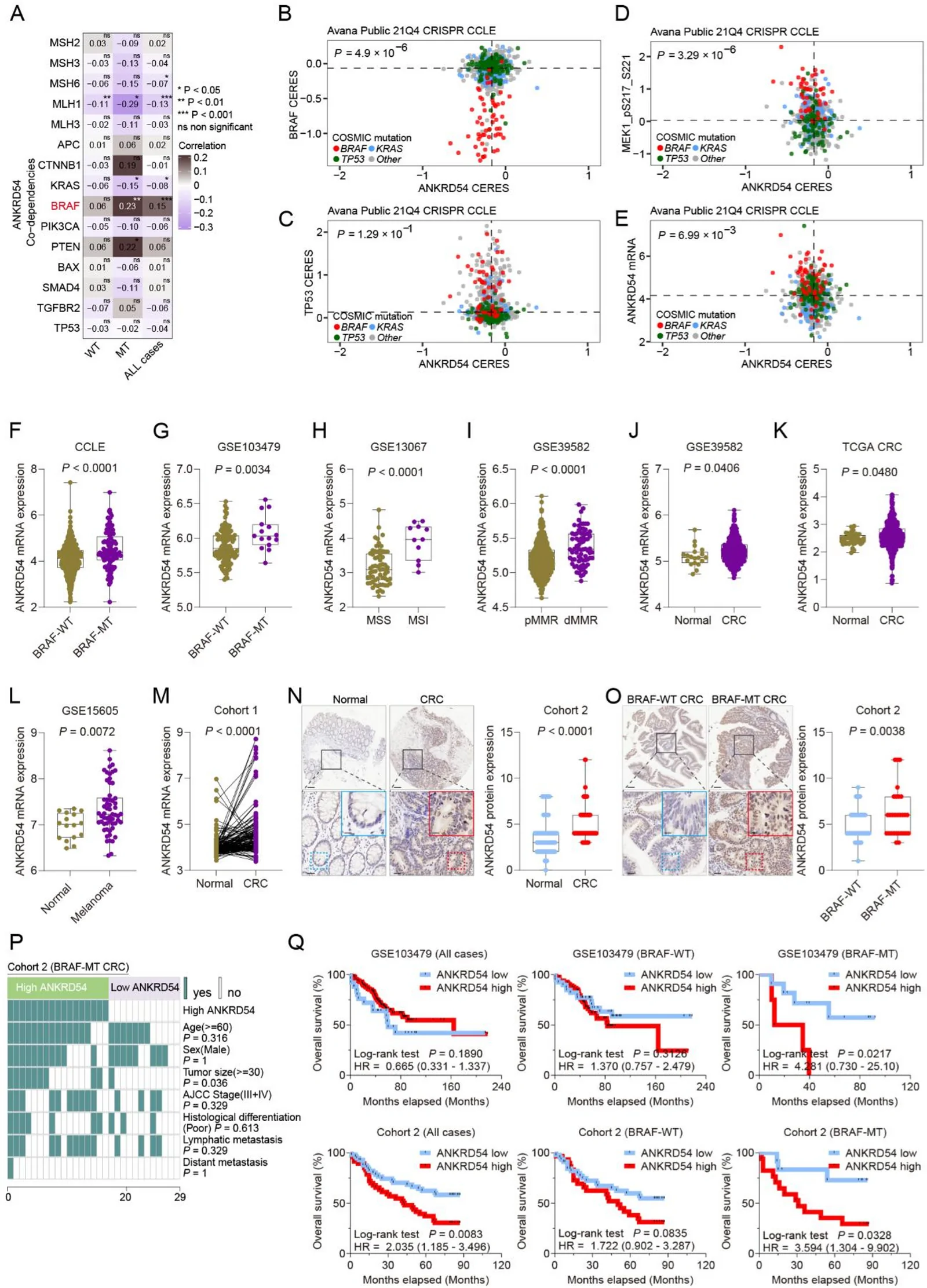
ANKRD54 serves as a prognostic indicator predicting poor outcomes in BRAF-MT CRC. **A** Analysis of the correlation between ANKRD54 genetic dependency (from DepMap CRISPR screens) and the mutation status of canonical CRC driver genes (MSH2, MSH3, MSH6, MLH1, MLH3, APC, CTNNB1, KRAS, BRAF, PIK3CA, PTEN, BAX, SMAD4, TGFBR2, TP53) in the CCLE dataset, as determined by Pearson’s correlation. **B-E** Scatter plot comparing ANKRD54 DepMap CERES scores to BRAF CERES scores (B), TP53 CERES scores (C), MEK1 (S217/S221) levels (D) and ANKRD54 mRNA (E) by COSMIC hotspot mutations to BRAF, KRAS and TP53. *P* values were calculated based on linear regression analysis. **F**,** G** Expression analysis of ANKRD54 mRNA levels in BRAF MT group versus BRAF WT group from CCLE pan-cancer (F) and GEO CRC (G) datasets (CCLE, *P* < 0.0001; GSE103479, *n* = 137, *P* = 0.0034). **H**,** I** Expression analysis of the ANKRD54 mRNA levels between MSI and MSS CRC in GEO database (GSE13067, *n* = 74, *P* < 0.0001) (H) and between pMMR and dMMR CRC (GSE39582, *n* = 519, *P* < 0.0001) (I). **J**,** K** Expression analysis of the ANKRD54 mRNA levels in normal and CRC tissues in GEO database (GSE39582, *n* = 585, *P* = 0.0406) (J) and TCGA database (*P* = 0.0480) (K). **L** Expression analysis of ANKRD54 mRNA levels in melanoma and normal tissues from GEO database (GSE15605, *n* = 74, *P* = 0.0072). **M** qRT-PCR determination of ANKRD54 mRNA levels in normal colorectal tissues and CRC tissues from Cohort 1 (*n* = 158, *P* < 0.0001). **N**,** O** Representative IHC staining images and statistical analysis of ANKRD54 in normal colorectal tissues and CRC tissues from Cohort 2 (*n* = 104, *P* < 0.0001) (N), and in BRAF WT CRC tissues and BRAF MT CRC tissues from Cohort 2 (*n* = 104, *P* = 0.0038) (O). Lower panels and upper-right of lower panels show successively enlarged images of indicated tissues. Scale bars = 100 μm (upper), 10 μm (lower) and 1 μm (upper-right of lower). **P** Comparison of clinicopathological characteristics between ANKRD54 high- and low-expression tumors in BRAF MT CRC patients from Cohort 2 (*n* = 29). **Q** Kaplan–Meier survival curves of OS in CRC patients in all cases (GSE103479, *n* = 156, *P* = 0.1890; Cohort 2, *n* = 104, *P* = 0.0083) or with WT BRAF (GSE103479, *n* = 122, *P* = 0.3126; Cohort 2, *n* = 75, *P* = 0.0835) and MT BRAF (GSE103479, *n* = 15, *P* = 0.0217; Cohort 2, *n* = 29, *P* = 0.0328) based on expression levels of ANKRD54. Data information: For (A), statistical significance was determined by analysis of the Pearson correlation coefficient. For (B-E), statistical significance was determined by the linear regression analysis. For (F-M, N, O), statistical significance was determined by the two-tailed Student’s t-test. For (P), statistical significance was determined by the Chi-square test. For (Q), statistical significance was determined by the log-rank test

CRC progression involves sequential alterations including MSI mediated by MSH2, MSH3, MSH6 [17, 18], dMMR driven by MLH1 and MLH3 [19, 20], tumor initiation mediated by APC and CTNNB1 [21], with subsequent progression promoted by oncogenes (KRAS, BRAF, and PIK3CA) [22], together with inactivating mutations in tumor suppressor genes (BAX, TGFBR2, TP53, SMAD4, PTEN) [23–26]. Against this established background, our analysis of single-gene CRISPR knockout screens from the DepMap and Cancer Cell Line Encyclopedia (CCLE) databases revealed that ANKRD54 knockout sensitivity strongly correlates with BRAF-MT status (Fig. 1A). Notably, in the small subset of BRAF-MT cell lines (~ 10%) exhibiting a dependency on ANKRD54 (Fig. 1B), which was independent of TP53 status but correlated with elevated MEK1 phosphorylation (Fig. 1C, D). This sensitive subgroup was further characterized by high ANKRD54 mRNA expression (Fig. 1E).

Examining CRC molecular subtypes, we observed an enrichment of ANKRD54 in BRAF-MT CRC. Bioinformatic analysis of the CCLE pan-cancer dataset and the GEO CRC dataset (Fig. 1F, G, S1A) confirmed that ANKRD54 expression was markedly higher in BRAF-MT context than in BRAF-WT. Given the established association between BRAF mutations and MSI, we further demonstrated that ANKRD54 was significantly upregulated in MSI CRC and, consequently, in dMMR CRC (Fig. 1H, I). Subsequently, we compared ANKRD54 expression levels between CRC and normal colorectal tissues. The GEO database revealed significant upregulation of ANKRD54 mRNA in CRC relative to normal colorectal tissues (Fig. 1J), which was replicated in the Cancer Genome Atlas (TCGA) database (Fig. 1K). Consistently, ANKRD54 was also elevated in melanoma compared with normal tissue, further supporting its oncogenic expression pattern (Fig. 1L). This upregulation was further corroborated at the transcriptional level in our Cohort 1 (Fig. 1M) and validated at the protein level by IHC in Cohort 2 (Fig. 1N). Immunohistochemical validation further confirmed that ANKRD54 protein expression was higher in BRAF-MT CRC than in the corresponding BRAF-WT cases (Fig. 1O).

Clinically, ANKRD54 upregulation showed clinicopathological significance only in BRAF-MT CRC, where higher expression correlated with increased tumor volume (≥ 30 cm³) (Fig. 1P). No significant associations between ANKRD54 and clinicopathological features were found in BRAF-WT patients (Fig. S1B). Survival analysis showed elevated ANKRD54 exclusively predicted unfavorable prognosis in BRAF-MT CRC (Fig. 1Q). This finding was supported by an analogous analysis in melanoma (Fig. S1C) and by TCGA data showing that ANKRD54 was associated with worse overall survival (OS) and progression-free survival (PFS) specifically in BRAF-MT tumors (Fig. S1D, E). To further validate ANKRD54 as an independent prognostic factor, we performed univariate analysis, which showed that both AJCC stage and ANKRD54 expression level were significantly associated with OS especially in BRAF-MT CRC patients (Fig. S1F, G). Multivariate analysis further demonstrated that, after adjusting for AJCC stage, high ANKRD54 expression remained an independent predictor of poor prognosis only in BRAF-MT CRC (Fig. S1H, I). These results establish ANKRD54 as a prognostic biomarker and underscore the need to explore its mechanistic role in BRAF-MT CRC.

### KAT2A-mediated H3K27 acetylation activates ANKRD54 transcription

**Fig. 2:**
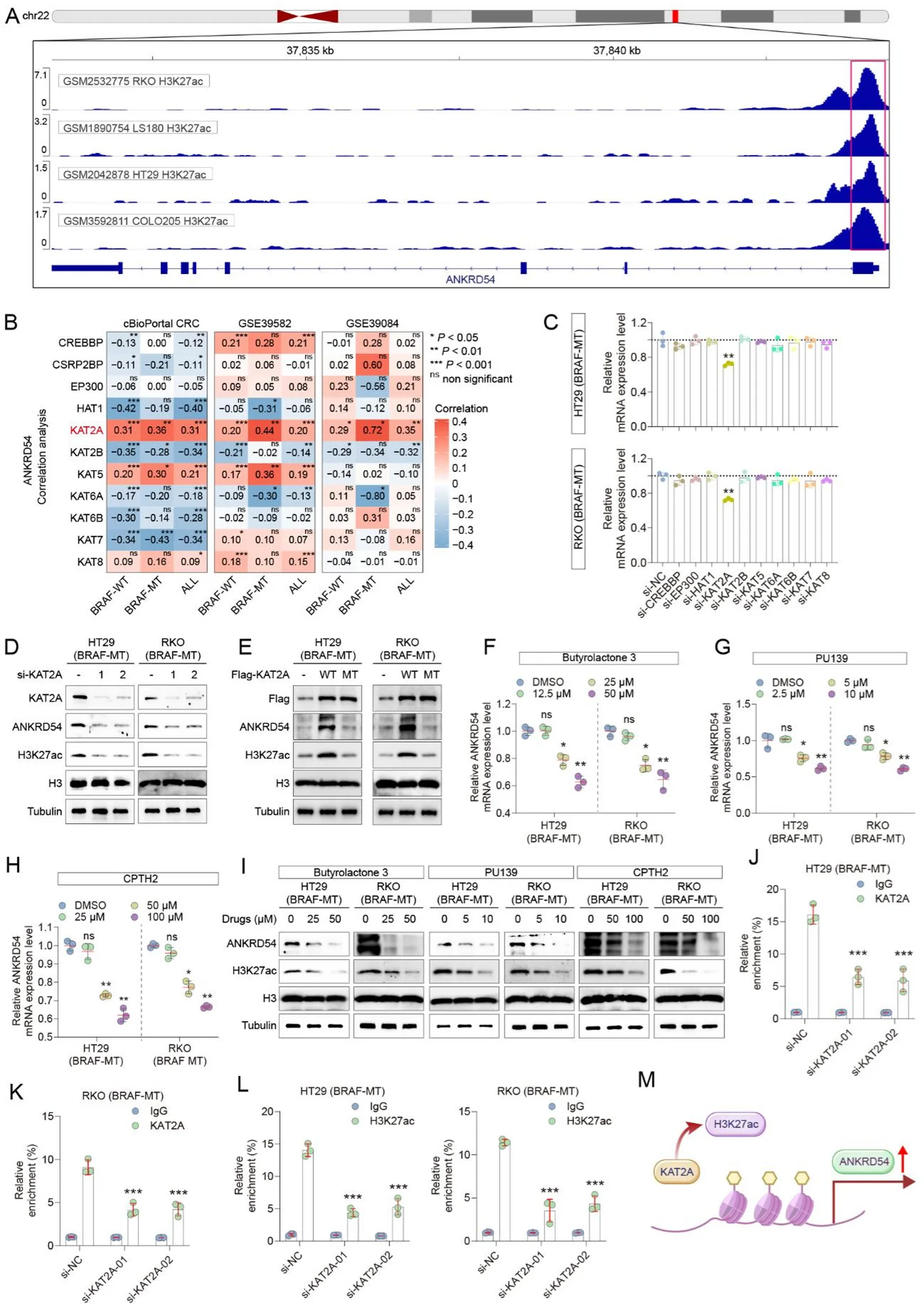
KAT2A-mediated H3K27 acetylation activates ANKRD54 transcription. **A** ChIP-seq data from the Cistrome DB website (http://cistrome.ora/db/) reveal a H3K27ac binding peak within the ANKRD54 promoter region. **B** Correlation analysis between ANKRD54 and reported H3K27ac-regulating genes (CREBBP, CSRP2BP, EP300, HAT1, KAT2A, KAT2B, KAT5, KAT6A, KAT6B, KAT7, KAT8) in CRC specimens from cBioPortal and GEO databases (GSE39582, GSE39084) (*P < 0.05, **P < 0.01, ***P < 0.001, ns = no significance). **C** qRT-PCR assays of ANKRD54 mRNA levels with respectively silencing reported H3K27ac-regulating genes in BRAF MT CRC cell lines (HT29, RKO) (biological replicates, *n* = 3; **P < 0.01, ns = no significance). **D** Western blot analysis of Flag-KAT2A, ANKRD54 and H3K27ac in BRAF MT (RKO and HT29) cells transfected with control siRNA or KAT2A siRNAs. **E** Western blot analysis of KAT2A and ANKRD54 in BRAF MT (RKO and HT29) cells with WT- or MT-Flag-KAT2A overexpression. **F-H** qRT-PCR assays of ANKRD54 mRNA levels in RKO and HT29 cells treated with KAT2A inhibitors Butyrolactone 3, PU139, and CPTH2 at indicated concentration (biological replicates, *n* = 3; *P < 0.05, **P < 0.01, ns = no significance). **I** Western blot analysis of ANKRD54 and H3K27ac in RKO and HT29 cells treated with KAT2A inhibitors Butyrolactone 3, PU139, and CPTH2 at indicated concentration. **J**,** K** ChIP-qRT-PCR assay verified the potential KAT2A-binding site in the ANKRD54 promoter region in RKO and HT29 cell lines transfected with control siRNA or KAT2A siRNAs. IgG was used as a control. Data are presented as mean ± SD (biological replicates, *n* = 3; ***P < 0.001). **L** ChIP-qRT-PCR assay verified the potential H3K27ac-binding site in the ANKRD54 promoter region in RKO and HT29 cell lines with control siRNA or KAT2A siRNAs. IgG was used as a control. Data are presented as mean ± SD (biological replicates, *n* = 3; ***P < 0.001). **M** Schematic diagram of the regulation relationship among KAT2A, H3K27ac and ANKRD54. Data information: For (B), statistical significance was determined by analysis of the Pearson correlation coefficient. For (C, F-H, J-L), statistical significance was determined by the two-tailed Student’s t-test

To identify molecular drivers of ANKRD54 overexpression in BRAF‑MT CRC, we first introduced recurrent BRAF mutations into cells, but this alone did not alter ANKRD54 expression (Fig. S2A). Subsequent bioinformatic analysis of chromatin immunoprecipitation sequencing (ChIP‑seq) revealed a peak of H3K27 acetylation (H3K27ac) at the ANKRD54 promoter in BRAF‑MT CRC cells (RKO, LS180, HT29 and COLO205) (Fig. 2A), suggesting histone acetylation as a potential regulatory mechanism.

To identify the upstream acetyltransferase regulating ANKRD54, we stratified colon cancer by ANKRD54 expression. This analysis revealed positive correlations with KAT2A, KAT5, and KAT8 (Fig. S2B), which were later corroborated in rectal cancer (Fig. S2C). Cross‑database correlation analyses using cBioPortal and GEO datasets showed that only KAT2A consistently correlated with ANKRD54 across all databases, with a notably stronger association in the BRAF-MT context (Fig. 2B). To functionally determine the primary regulator of H3K27ac at the ANKRD54 promoter, we silenced these candidate acetyltransferases individually [27, 28]. Only KAT2A suppression reduced ANKRD54 mRNA in BRAF‑MT CRC cells (Fig. 2C). Interestingly, overexpression of different BRAF mutants and WT-BRAF did not alter KAT2A expression (Fig. S2D). At the protein level, KAT2A inhibition downregulated H3K27ac and subsequently ANKRD54, specifically in BRAF‑MT but not BRAF‑WT CRC (Fig. 2D, S2E). Meanwhile, WT-Flag-KAT2A, but not the MT-Flag-KAT2A (HAT-dead mutant E575A/D615A) [29], promoted ANKRD54 expression accompanied by enhanced H3K27ac in BRAF-MT CRC cells (Fig. 2E). This causal relationship was further supported by dose‑dependent reductions in ANKRD54 mRNA (Fig. 2F-H) and protein (Fig. 2I) upon KAT2A inhibitor treatment. Importantly, this regulatory axis was absent in BRAF‑WT CRC cells, underscoring its context‑specificity (Fig. S2E-H).

ChIP assays confirmed enrichment of ANKRD54 promoter fragments in KAT2A‑immunoprecipitated DNA compared to IgG controls, which was markedly reduced upon KAT2A knockdown (Fig. 2J, K). To link this to enzymatic function, we measured H3K27ac levels at the same locus. As expected, ChIP assays showed high H3K27ac signal at the ANKRD54 promoter, and this signal was directly dependent on KAT2A, as its silencing attenuated H3K27ac enrichment (Fig. 2L). Together, these data showed KAT2A-mediated H3K27ac at the ANKRD54 promoter drives its transcriptional upregulation specifically in BRAF-MT CRC (Fig. 2M).

### Targeting ANKRD54 suppresses malignancy of BRAF-MT CRC

**Fig. 3:**
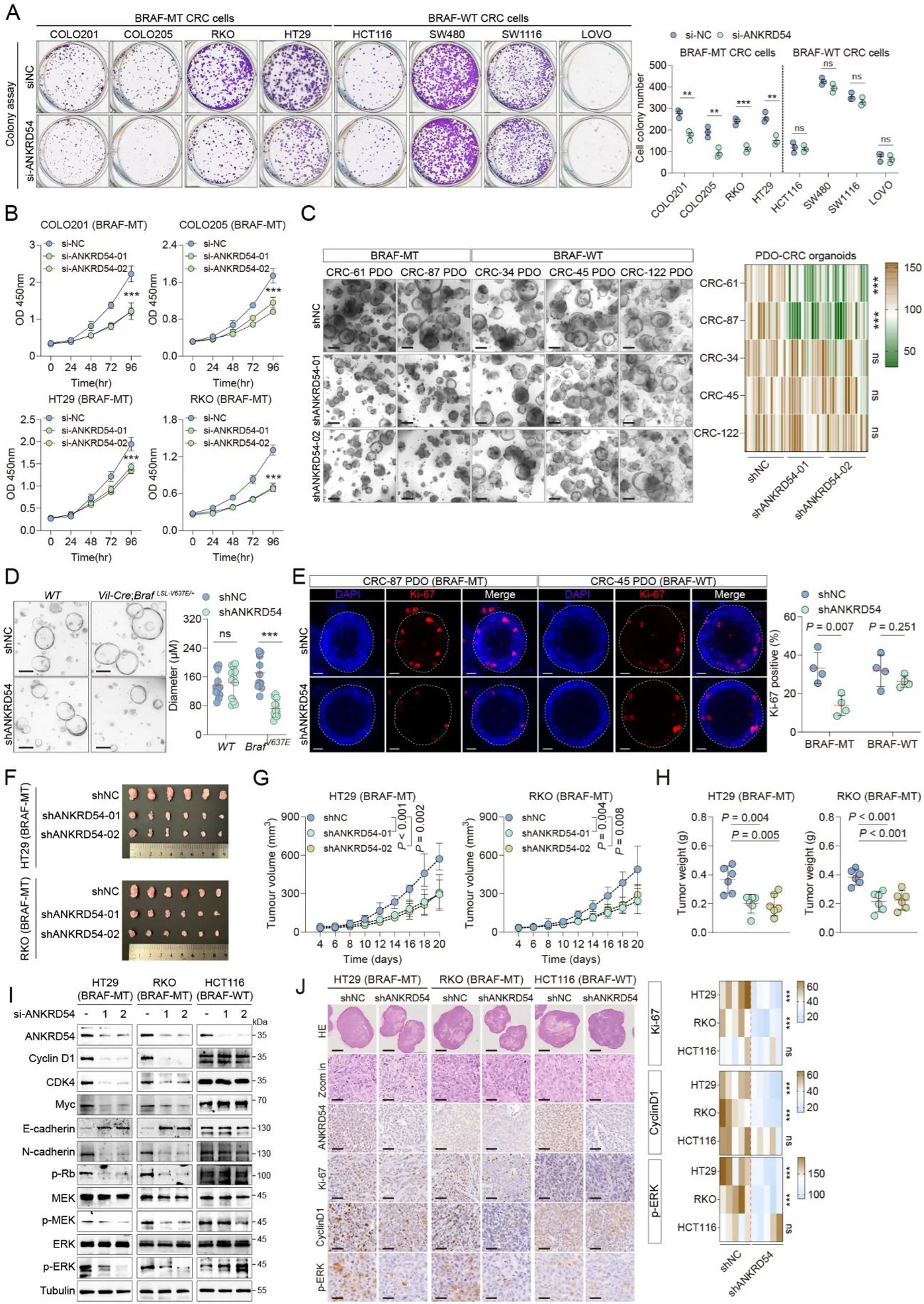
Targeting ANKRD54 suppresses malignancy of BRAF-MT CRC. **A** Colony formation assay was performed in BRAF MT CRC (COLO201, COLO205, HT29, RKO) and BRAF WT CRC (HCT116, SW480, SW1116, LOVO) cell lines transfected with si-NC and si-ANKRD54. Data are presented as mean ± SD (biological replicates, *n* = 3, ns = no significance, **P < 0.01). **B** Cell viability assay was performed in BRAF MT CRC cell lines (COLO201, COLO205, HT29, RKO) transfected with si-NC or si-ANKRD54s. Data are presented as mean ± SD (biological replicates, *n* = 5, ***P < 0.001). **C** Bright-field microscopy images of BRAF MT PDOs (CRC-61 and CRC-87) and BRAF WT PDOs (CRC-34, CRC-45 and CRC-122) after ANKRD54 inhibition. Organoid diameters were measured using ImageJ (Scale bar = 100 μm, ***P < 0.001, ns = no significance). **D** Bright-field microscopy images of organoid derived from C57BL/6 mice colorectal tissues and Vil-CRE; BRAF^LSL−V637E/+^ mice colorectal tissues after ANKRD54 inhibition. Organoid diameters were measured using ImageJ (Scale bar = 100 μm, ***P < 0.001, ns = no significance). **E** Representative confocal images showing Ki-67 and DAPI staining in CRC-87 PDO and CRC-45 PDO transfected with control vector and shANKRD54 vector (Scale bar = 25 μm). Ki-67 (Red) and DAPI (Blue). Positive rates of Ki-67 staining were calculated and compared from 10 random fields per sample (BRAF-MT, *P* = 0.007; BRAF-WT, *P* = 0.251). **F-H** Representative images of tumors and analysis in nude mice intervened with shNC or shANKRD54 BRAF MT (RKO and HT29) cells. The stripped tumors are shown (F) and growth curve was plotted (G). The tumors were extracted and weighted after 20 days (H). Data are presented as mean ± SD (biological replicates, *n* = 6). **I** Western blot analysis of ANKRD54, Cyclin D1, CDK4, Myc, E-cadherin, N-cadherin, p-Rb, MEK, p-MEK, ERK and p-ERK in HT29, RKO and HCT116 cells after silencing ANKRD54. **J** Representative HE and IHC staining images and quantitative analysis of ANKRD54, Ki-67, CyclinD1 and p-ERK in subcutaneous tumors from nude mice in Fig. 3F and S3E. Scale bars of HE = 500 μm, Scale bars of magnification and IHC staining images = 25 μm. Data are presented as mean ± SD (biological replicates, *n* = 5; ns = no significance, ***P < 0.001). Data information: For (A-E, G, H), statistical significance was determined by the two-tailed Student’s t-test. For (J), statistical significance was determined by the nonparametric Mann-Whitney test

Although the ANKRD family shares a conserved ankyrin repeat structure, its members differ in genomic location, expression, and function. To elucidate their roles in CRC, we screened the ANKRD family in CRC cell lines using the DepMap database and found that BRAF‑MT cells were preferentially dependent on ANKRD54, making it the top candidate (Fig. S3A). To functionally test this association, we silenced ANKRD54 in multiple BRAF-MT cell lines and found impaired malignant properties, including colony formation, proliferation, invasion, and migration, whereas BRAF-WT cells were unaffected (Fig. 3A, B, S3B-D).

ANKRD54 inhibition also markedly stunted the growth of BRAF‑MT patient‑derived organoids (PDOs) while leaving BRAF‑WT PDOs unaffected (Fig. 3C). This phenotype was recapitulated in organoids from a conditional knock‑in (cKI) mouse model (Fig. 3D). The growth arrest in BRAF‑MT CRC was associated with reduced proliferative activity, as shown by decreased Ki‑67‑positive cells (Fig. 3E). In vivo, xenograft models demonstrated that ANKRD54 suppression significantly inhibited BRAF‑MT tumor growth, reducing both volume and weight (Fig. 3F-H). This anti‑tumor activity was selective, with no effect observed in xenografts derived from BRAF‑WT CRC cells (Fig. S3E-G).

ANKRD54 knockdown in BRAF-MT cells suppressed cell-cycle regulators (cyclin D1, CDK4, MYC, p-Rb), reversed epithelial-mesenchymal transition markers (increased E-cadherin, decreased N-cadherin), and attenuated MAPK signaling (reduced pMEK, pERK) (Fig. 3I); these changes were corroborated by IHC in xenograft tumors (Fig. 3J). These results demonstrate that targeting ANKRD54 suppresses malignant progression in BRAF-MT CRC.

### ANKRD54 manipulates FA metabolism by CEBPB-mediated upregulation of ELOVL6

**Fig. 4:**
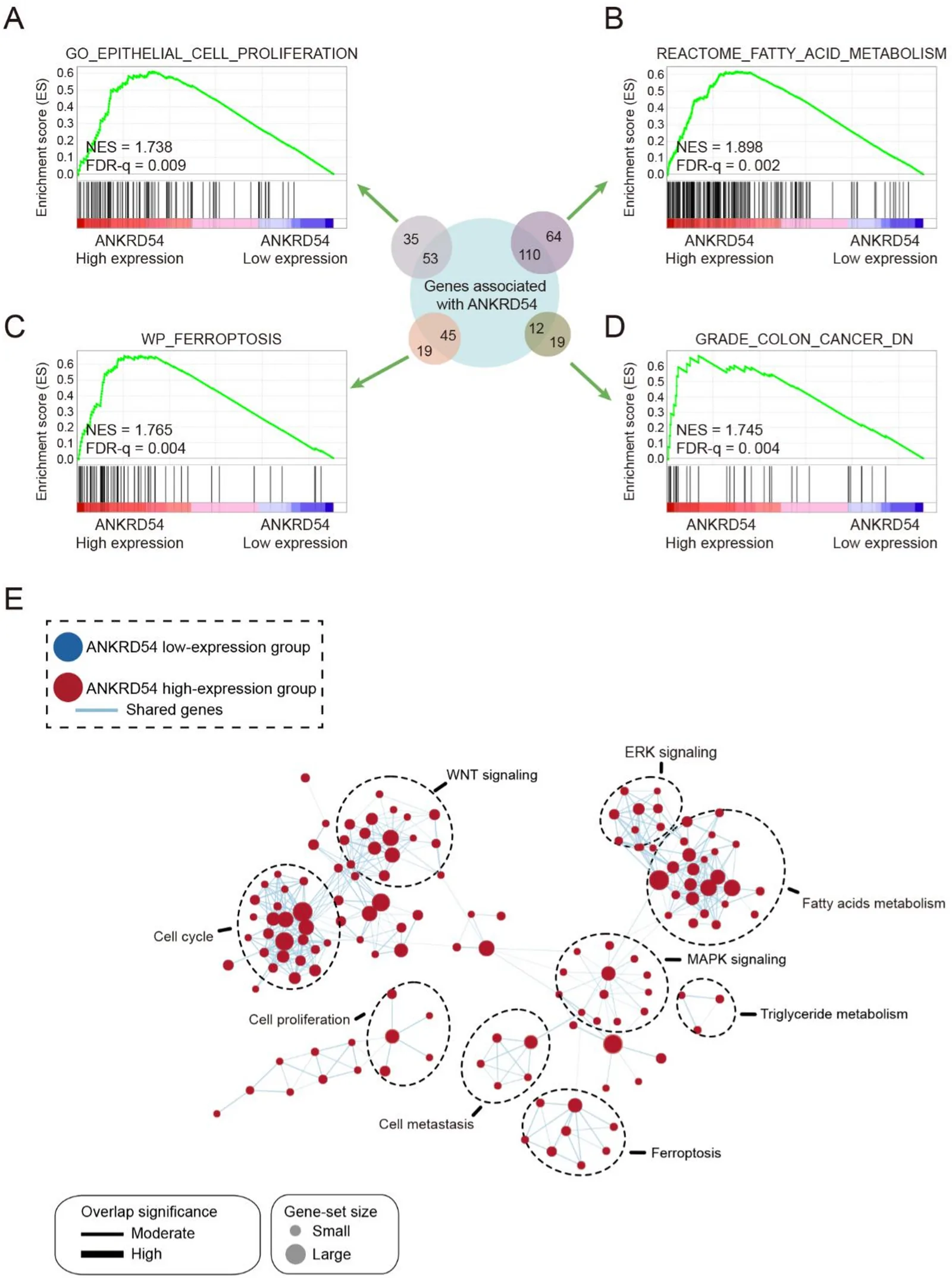
ANKRD54 manipulates FA metabolism by CEBPB-mediated upregulation of ELOVL6. **A-D** Overview of GSEA identified the differential gene profiles in ANKRD54 low-expression and ANKRD54 high-expression cells by TCGA CRC cohort. The sum of the numbers in the small circles of different colors is the total number of genes in each gene sets related to cell proliferation, fatty acid metabolism, ferroptosis and CRC specific signature. The number where the two circles intersect is the total number of genes that are both associated with ANKRD54 and belong to the gene set related to different signatures. **E** TCGA comparison between patients with ANKRD54 low expression (red) and ANKRD54 high expression (blue). Median split, *n* = 319 (TCGA CRC). Distinct pathways and biological processes were delineated between the two patient groups. Cystoscope and Enrichment map were used for visualization of the TCGA results (1% FDR; *P* = 0.005). Each node represents an enriched gene set, grouped by similarity

To elucidate the molecular consequences of ANKRD54 expression in BRAF-MT CRC, we performed Gene Set Enrichment Analysis (GSEA) on the TCGA CRC cohort. Patients with high ANKRD54 expression showed strong enrichment of gene signatures related to cancer progression, including proliferation (GO_EPITHELIAL_CELL_PROLIFERATION), fatty acid metabolism (REACTOME_FATTY_ACID_METABOLISM), ferroptosis (WP_FERROPTOSIS), and CRC-specific pathways (GRADE_COLON_CANCER_DN) (Fig. 4A-D). Other tumorigenic gene sets similarly correlated with ANKRD54 expression (Fig. S4A-F). Cluster analysis confirmed that high ANKRD54 expression was associated with gene sets involved in cell cycle, proliferation, WNT/MAPK/ERK signaling, metastasis, FA/triglyceride (TG) metabolism, and ferroptosis (Fig. 4E). These bioinformatic findings suggest that ANKRD54 links malignant programs to FA metabolism and ferroptosis in BRAF-MT CRC.

**Fig. 5:**
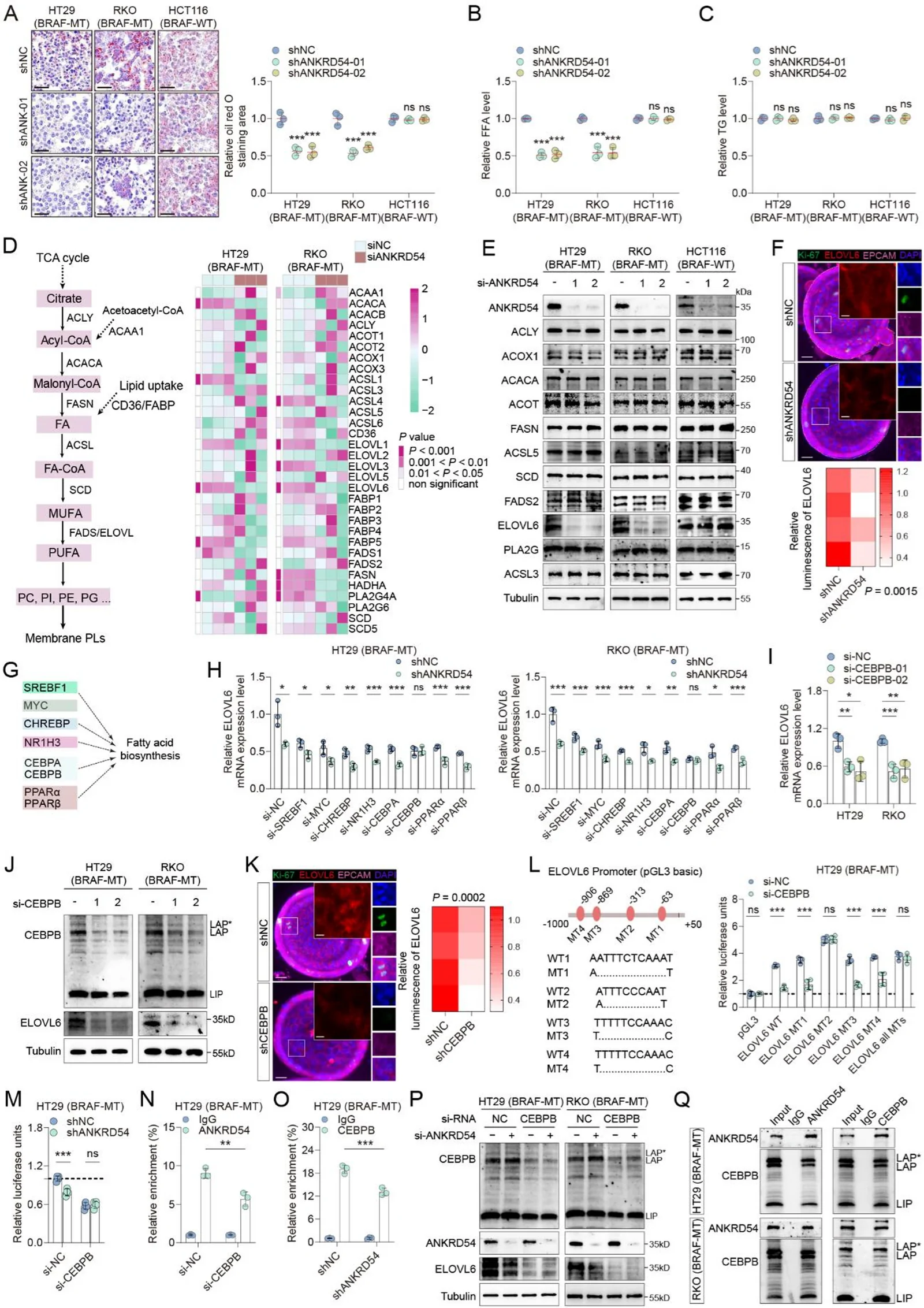
ANKRD54 manipulates FA metabolism by CEBPB-mediated upregulation of ELOVL6. **A** Oil Red O staining in BRAF MT CRC (HT29, RKO) and BRAF WT CRC (HCT116) cell lines following ANKRD54 knockdown. Scale bar = 20 μm (ns = no significance, ***P < 0.001). **B**,** C** Measurements analyses of FFA (B) and TG (C) in BRAF MT CRC (HT29, RKO) and BRAF WT CRC (HCT116) cell lines with stable ANKRD54 knockdown clones (biological replicates, *n* = 3; ns = no significance, ***P < 0.001). **D** mRNA expression levels of 32 FA metabolism-related genes were evaluated using qRT–PCR in ANKRD54 inhibitor transfected BRAF MT CRC cell lines (RKO and HT29) (biological replicates, *n* = 3). **E** Western blot analysis of ANKRD54, ACLY, ACOX1, ACACA, ACOT, FASN, ACSL5, SCD, FADS2, ELOVL6, PLA2G and ACSL3 in HT29, RKO and HCT116 cells after silencing ANKRD54. **F** Representative confocal images showing Ki-67, ELOVL6, EPCAM and DAPI staining in CRC-87 PDO transfected with control vector and shANKRD54 vector (Scale bar = 50 μm). Ki-67 (Green), ELOVL6 (Red), EPCAM (Pink) and DAPI (Blue). Luminescence of ELOVL6 staining was calculated and compared from 10 random fields per sample (*P* = 0.0015). **G** 8 reported candidate genes of FA biosynthesis (SREBF1, MYC, CHREBP, NR1H3, CEBPA, CEBPB, PPARα, and PPARβ). **H** qRT-PCR determination of ELOVL6 mRNA levels in ANKRD54-knockdown HT29 and RKO cells following 8 candidate genes separately silenced (biological replicates, *n* = 3; *P < 0.05, **P < 0.01, ***P < 0.001, ns = no significance). **I** qRT-PCR determination of ELOVL6 mRNA levels in CEBPB-silencing HT29 and RKO cells (biological replicates, *n* = 3; *P < 0.05, **P < 0.01, ***P < 0.001). **J** Western blot analysis of ELOVL6 protein levels in RKO and HT29 cells following CEBPB silenced. **K** Representative confocal images showing Ki-67, ELOVL6, EPCAM and DAPI staining in CRC-87 PDO transfected with control vector and shCEBPB vector (Scale bar = 50 μm). Ki-67 (Green), ELOVL6 (Red), EPCAM (Pink) and DAPI (Blue). Luminescence of ELOVL6 staining was calculated and compared from 10 random fields per sample (*P* = 0.0002). **L** Left-Upper: Schematic diagram of WT and deletion mutants (MT1: -63 bp; MT2: -313 bp; MT3: -869 bp; MT4: -906 bp) of ELOVL6 promoter-containing fragments with putative CEBPB-binding sites. Left-Lower: Base sequence at the mutation site (WT1: AATTTCTCAAAT, MT1: A………T; WT2: ATTTCCCAAT, MT2: A………T; WT3: TTTTTCCAAAC, MT3: T………C; WT4: TTTTTCCAAAC, MT4: T………C). Transcription factor binding site predictions were conducted via JASPAR (https://jaspar.elixir.no/). Right: Luciferase activity in HT29 cells with CEBPB knockdown following transfection with ELOVL6 promoter luciferase reporter vectors (biological replicates, *n* = 4; ***P < 0.001, ns = no significance). **M** Luciferase activity in HT29 cells with ANKRD54 knockdown following transfection with control siRNA, CEBPB siRNAs and ELOVL6 WT promoter luciferase reporter vectors (biological replicates, *n* = 4; ***P < 0.001, ns = no significance). **N**,** O** ChIP–qRT–PCR assays verified the potential ANKRD54 (N)-binding interaction and CEBPB (O) -binding site in the ELOVL6 promoter region in HT29 cells after inhibition of CEBPB (N) and ANKRD54 (O). IgG was used as a control (biological replicates, *n* = 3; **P < 0.01, ***P < 0.001). **P** Western blot analysis of CEBPB (including three isoforms LAP^*^, LAP and LIP), ANKRD54 and ELOVL6 in HT29 and RKO treated with CEBPB or ANKRD54 siRNAs. **Q** Co-immunoprecipitation and western blotting showing the mutual binding of ANKRD54 with CEBPB in RKO and HT29 cells, representative of three independent experiments. Data information: For (A-D, H, I, L-O), statistical significance was determined by the two-tailed Student’s t-test. For (F, K), statistical significance was determined by the nonparametric Mann-Whitney test

To validate the link between ANKRD54 and FA metabolism, we first assessed lipid content. ANKRD54 knockdown markedly reduced lipid droplets in BRAF-MT cells, as shown by Oil Red O staining (Fig. 5A). Quantification revealed a selective decrease in free fatty acids (FFAs), while TG levels remained unchanged (Fig. 5B, C). We next sought to identify the downstream effector of ANKRD54 among key FA metabolism genes. Following ANKRD54 silencing in BRAF-MT cells, mRNA levels of both ELOVL6 and PLA2G4A were altered. ELOVL6 was consistently downregulated in HT29 and RKO cells, whereas PLA2G4A exhibited opposite trends (Fig. 5D). Subsequent Western blot analysis confirmed that only ELOVL6 protein levels were correspondingly reduced (Fig. 5E). This regulatory axis was further validated in PDOs, where ANKRD54 suppression decreased both ELOVL6 and Ki-67 specifically in BRAF-MT samples (Fig. 5F, S5A, B).

To identify the intermediary linking ANKRD54 to ELOVL6, we performed correlation analysis between ANKRD54 and putative ELOVL6 transcription factors [30–34], identifying CEBPB as the top candidate (Fig. 5G, S5C). qRT-PCR further confirmed that CEBPB positively regulates ELOVL6 transcription and that ANKRD54-dependent regulation of ELOVL6 requires CEBPB expression (Fig. 5H). Accordingly, CEBPB inhibition downregulated ELOVL6 at both mRNA and protein levels (Fig. 5I, J). In BRAF-MT PDOs, CEBPB knockdown similarly reduced ELOVL6 expression with decreased Ki-67 positivity (Fig. 5K, S5D, E). Functionally, CEBPB knockdown impaired organoid formation in both BRAF-MT PDOs and organoids from cKI mouse models (Fig. S5F, G). To dissect the precise mechanism of the ANKRD54/CEBPB/ELOVL6 axis, we screened the JASPAR database for potential CEBPB binding sites on the ELOVL6 promoter and confirmed MT2 as the key functional site using luciferase assays (Fig. 5L, S5H). Importantly, ANKRD54 knockdown attenuated ELOVL6 promoter activity, an effect that was completely abolished upon CEBPB silencing (Fig. 5M, S5I).

Having established CEBPB as the key downstream effector required for ANKRD54-dependent ELOVL6 transcription, we next investigated the potential regulatory mechanisms as proteolysis, karyotropism, and transactivation. ANKRD54 knockdown did not alter CEBPB protein degradation in cycloheximide chase assays (Fig. S5J, K), and CEBPB nuclear localization remained mainly unchanged irrespective of ANKRD54 expression levels (Fig. S5L). Correspondingly, dual-luciferase assay suggested that ANKRD54 markedly increased the transactivation capacity of CEBPB (Fig. S5M). Correspondingly, ChIP assays showed CEBPB silencing reduced ANKRD54 recruitment to the ELOVL6 promoter, and ANKRD54 knockdown diminished CEBPB enrichment (Fig. 5N, O, S5N). ANKRD54 inhibition failed to suppress ELOVL6 in CEBPB-silenced cells (Fig. 5P), and co-immunoprecipitation (Co-IP) demonstrated direct ANKRD54-CEBPB interaction (Fig. 5Q). These data delineate a cascade where ANKRD54 interacts with CEBPB to enhance its ELOVL6 promoter recruitment and drive expression.

### ANKRD54/ELOVL6 axis suppresses ferroptosis in BRAF-MT CRC

**Fig. 6:**
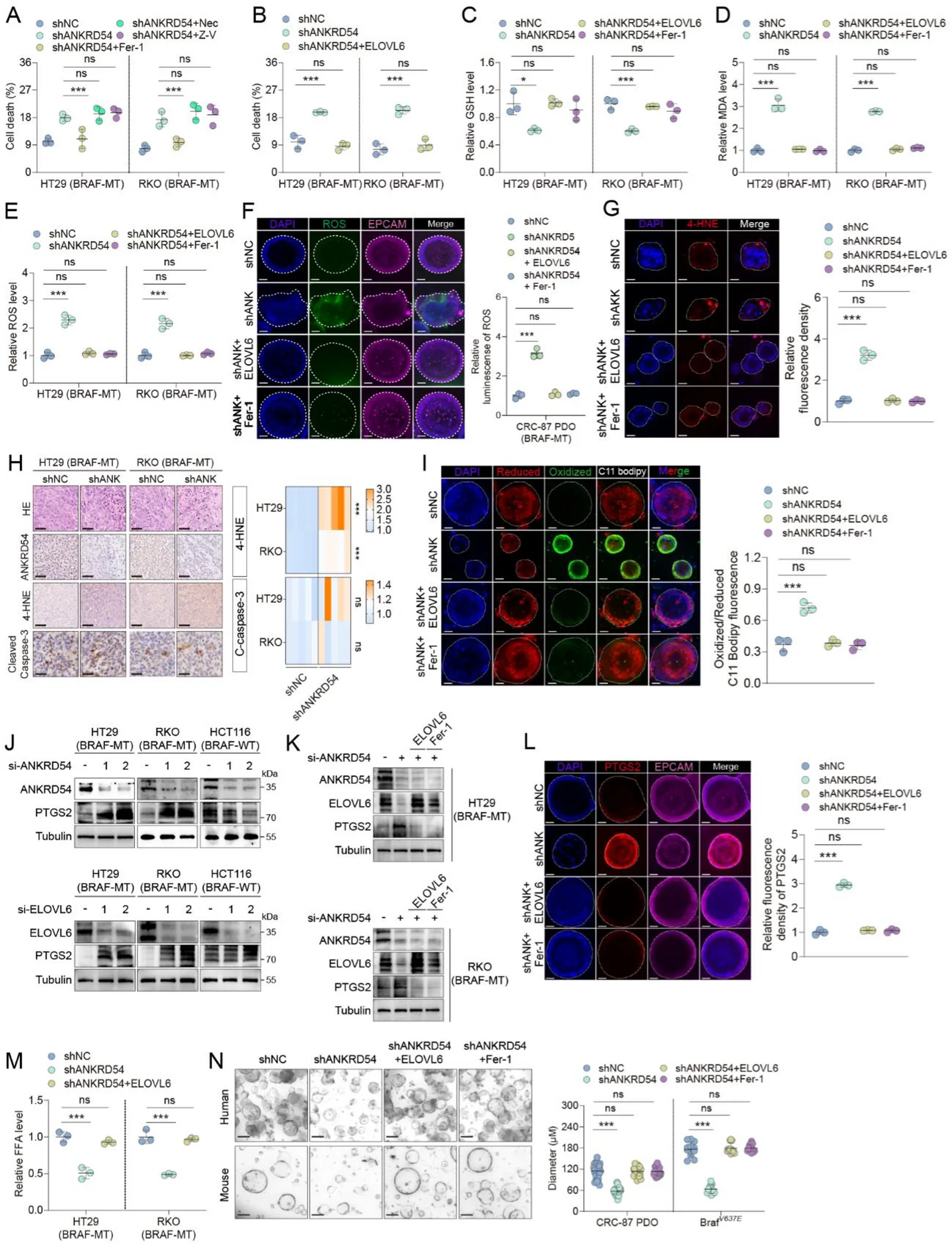
ANKRD54/ELOVL6 axis suppresses ferroptosis in BRAF-MT CRC. **A**,** B** Cell viability assay following ANKRD54 knockdown and treated with Nec, Fer-1, Z-V (A) and overexpression of ELOVL6 (B). Ratio of dead cells in HT29 and RKO cells was measured by trypan blue staining. Data are mean ± SD (biologically independent samples, *n* = 3, ***P < 0.001, ns = no significance). **C**,** D** The GSH levels (C) in HT29 and RKO cells following ANKRD54 knockdown and treated with Fer-1 or overexpression of ELOVL6 were measured by GSH assay kit. The MDA levels (D) were measured by Thio barbituric acid (TBA) colorimetric assay (biologically independent samples, *n* = 3, *P < 0.05, ***P < 0.001, ns = no significance). **E** The ROS levels in HT29 and RKO cells following ANKRD54 knockdown and treated with Fer-1 or overexpression of ELOVL6 were measured by GSH assay kit. The ROS levels were measured by flow cytometry analysis of CM-H2DCFDA staining (biologically independent samples, *n* = 3, ***P < 0.001, ns = no significance). **F** Representative confocal images showing ROS, EPCAM, and DAPI staining in PDO-CRC-87 following ANKRD54 knockdown and treated with Fer-1 or overexpression of ELOVL6 (Scale bar = 50 μm). ROS (Green), EPCAM (Pink) and DAPI (Blue). Luminescence of ROS staining was calculated and compared from 10 random fields per sample (***P < 0.001, ns = no significance). **G** Representative confocal images showing 4-HNE and DAPI staining in PDO-CRC-87 following ANKRD54 knockdown and treated with Fer-1 or overexpression of ELOVL6 (Scale bar = 50 μm). 4-HNE (Red) and DAPI (Blue). Luminescence of 4-HNE staining was calculated and compared from 10 random fields per sample (***P < 0.001, ns = no significance). **H** HE, IHC stains and statistical analysis of 4-HNE and cleaved caspase-3 of HT29 and RKO cells xenograft in nude mice (Scale bar = 25 μm, ***P < 0.001, ns = no significance). **I** Representative images of PDO-CRC-87 following ANKRD54 knockdown and treated with Fer-1 or overexpression of ELOVL6, stained with DAPI (blue) and C11-BODIPY (Red, reduced form of C11-BODIPY; Green, oxidized form of C11-BODIPY). Scale bar = 50 μm. Oxidized/Reduced C11 Bodipy fluorescence was calculated and compared from 10 random fields per sample (***P < 0.001, ns = no significance). **J** Western blot analysis of ANKRD54, ELOVL6 and PTGS2 in HT29, RKO and HCT116 cells treated with ANKRD54 or ELOVL6 siRNAs. **K** Western blot analysis of ANKRD54, ELOVL6 and PTGS2 in RKO and HT29 cells treated with ANKRD54 siRNA and Fer-1 or overexpression of ELOVL6. **L** Representative confocal images showing PTGS2, EPCAM, and DAPI staining in PDO-CRC-87 transfected with shANKRD54 vector and treated with Fer-1 or overexpression of ELOVL6 (Scale bar = 50 μm). PTGS2 (Red), EPCAM (Pink) and DAPI (Blue). Luminescence of PTGS2 staining was calculated and compared from 10 random fields per sample (***P < 0.001, ns = no significance). **M** Measurements analyses of FFAs in HT29 and RKO cells with ANKRD54 knockdown and overexpression of ELOVL6 (biological replicates, *n* = 3; ns = no significance, ***P < 0.001). **N** Bright-field microscopy images of CRC-87 PDO and organoid derived from Vil-CRE; BRAF^LSL−V637E/+^ mice colorectal tissues with ANKRD54 knockdown and treated with Fer-1 or overexpression of ELOVL6. Organoid diameters were measured using ImageJ (Scale bar = 100 μm, ***P < 0.001, ns = no significance). Data information: For (A-E, M, N), statistical significance was determined by the two-tailed Student’s t-test. For (F-I, L), statistical significance was determined by the nonparametric Mann-Whitney test

To elucidate how the ANKRD54/ELOVL6 axis supports malignant growth in BRAF‑MT CRC, we knocked down ANKRD54 or ELOVL6 and observed significantly increased cell death specifically in BRAF‑MT cells (Fig. S6A, B). This cell death was identified as ferroptosis, since it was rescued by the ferroptosis inhibitor Fer‑1 but not by inhibitors of apoptosis (Z-V) or necroptosis (Fig. 6A). Concurrently, ELOVL6 overexpression abrogated the cell death induced by ANKRD54 knockdown (Fig. 6B).

We next examined the core biochemical features of ferroptosis. ANKRD54 depletion compromised the antioxidant system, as evidenced by reduced intracellular glutathione (GSH), which was restored by Fer-1 treatment or ELOVL6 overexpression (Fig. 6C, S6C, D). Consistently, lipid peroxidation was markedly increased, as shown by elevated malondialdehyde (MDA), and this effect was similarly reversed by Fer-1 or ELOVL6 overexpression (Fig. 6D, S6E, F). ROS levels were also dramatically increased, indicating pronounced oxidative stress (Fig. 6E, S6G, H). In BRAF-MT PDOs, knockdown of either ANKRD54 or ELOVL6 likewise increased ROS levels, which were rescued by Fer-1 or ELOVL6 overexpression (Fig. 6F and S6I). In parallel, ANKRD54 or ELOVL6 knockdown also increased 4-HNE levels, and this effect was similarly reversed by Fer-1 or ELOVL6 overexpression (Fig. 6G and S6J). In xenografts, ANKRD54 inhibition increased 4-HNE staining without affecting cleaved caspase-3 (Fig. 6H). Direct visualization with C11-BODIPY further confirmed enhanced lipid peroxidation after ANKRD54 or ELOVL6 loss in BRAF-MT CRC, an effect that was similarly rescued by Fer-1 or ELOVL6 overexpression (Fig. 6I, S6K). PTGS2 was upregulated following ANKRD54 or ELOVL6 silencing in BRAF-MT cells, while other ferroptosis regulators remained unchanged (Fig. 6J, S6L, M). This upregulation was abrogated by ELOVL6 overexpression or Fer-1 treatment (Fig. 6K) and was similarly confirmed in PDOs by immunostaining (Fig. 6L, S6N).

Finally, we linked these observations to metabolic function and malignant phenotype. The reduction in FFA levels caused by ANKRD54 suppression was fully restored by ELOVL6 overexpression (Fig. 6M), while ELOVL6 inhibition alone impaired the formation of BRAF‑MT organoids (Fig. S6O, P). Notably, the organoid formation defect induced by ANKRD54 inhibition was effectively rescued by either restoring ELOVL6 expression or treating with Fer‑1 (Fig. 6N). Taken together, these results provide comprehensive evidence that the ANKRD54/ELOVL6 axis critically regulates cell proliferation in BRAF‑MT CRC, primarily by modulating FA metabolism and suppressing ferroptosis.

### Therapeutic targeting of ANKRD54/ELOVL6 axis synergizes with BRAF^V600E^ inhibitor

**Fig. 7:**
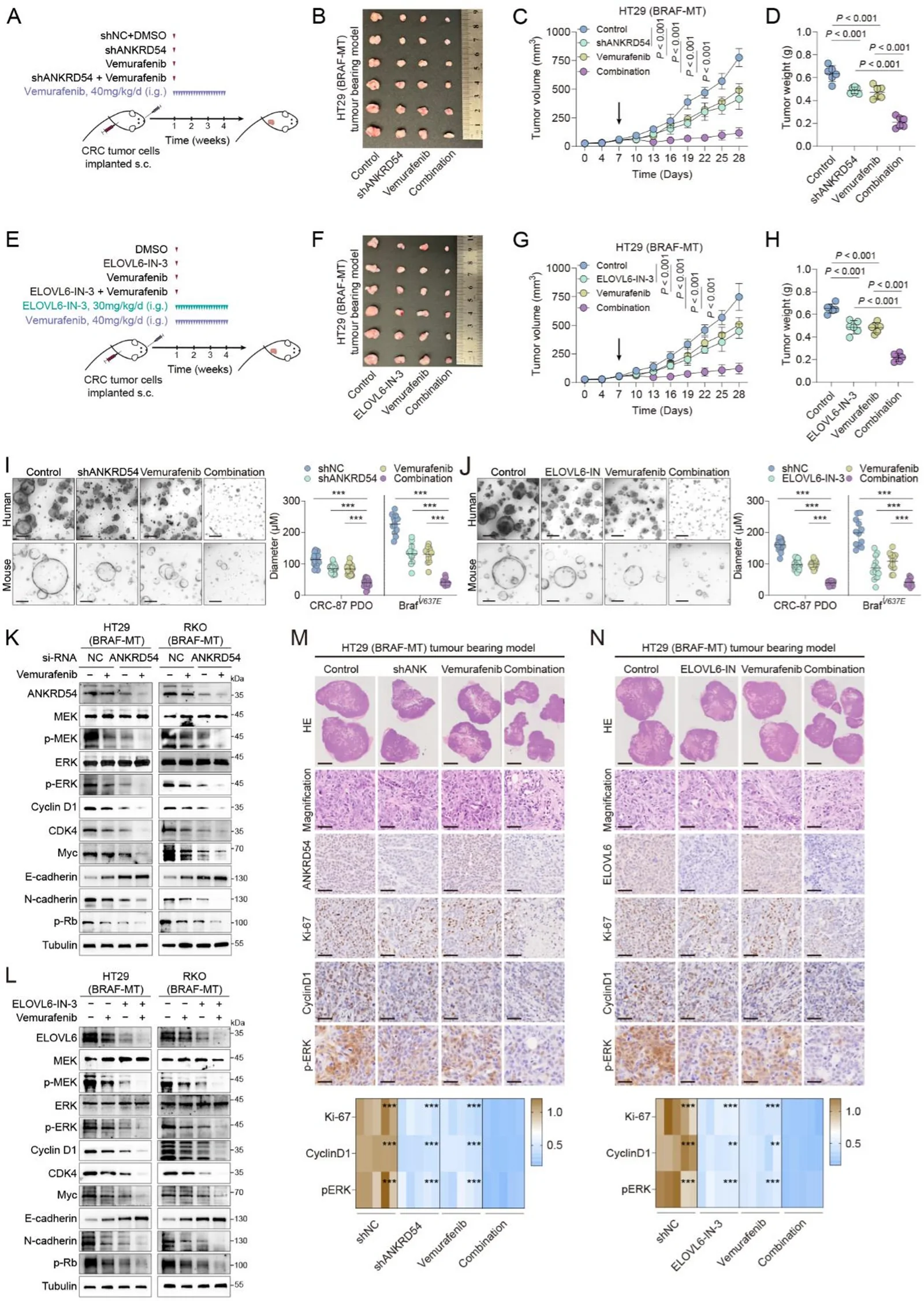
Therapeutic targeting of ANKRD54/ELOVL6 axis synergizes with BRAF^V600E^ inhibitor. **A**,** E** Schematic diagram of the CRC mouse model: shANKRD54 vectors were intratumorally injected (A) or ELOVL6-IN-3 was administered by oral gavage (30 mg/kg/day) (E), while Vemurafenib was administered by oral gavage (40 mg/kg/day), either as monotherapies or in combination. **B-D** Representative images of tumors and analysis in nude mice intervened with shNC, shANKRD54, Vemurafenib and combination of shANKRD54 and Vemurafenib. The stripped tumors are shown (B) and growth curve was plotted (C). The tumors were extracted and weighted after 28 days (D). Data are presented as mean ± SD (biological replicates, *n* = 6, *P <* 0.001). **F-H** Representative images of tumors and analysis in nude mice intervened with control, ELOVL6-IN-3, Vemurafenib and combination of ELOVL6-IN-3 and Vemurafenib. The stripped tumors are shown (F) and growth curve was plotted (G). The tumors were extracted and weighted after 28 days (H). Data are presented as mean ± SD (biological replicates, *n* = 6, *P <* 0.001). **I**,** J** Bright-field microscopy images of CRC-87 PDO and organoid derived from Vil-CRE; BRAF^LSL−V637E/+^ mice colorectal tissues with shNC, shANKRD54 (I) or ELOVL6-IN-3 (J), Vemurafenib and combination. Organoid diameters were measured using ImageJ (Scale bar = 100 μm, ***P < 0.001). **K**,** L** Western blot analysis of ANKRD54 (K), ELOVL6 (L), MEK, р-MEK, ERK, p-ERK, CyclinD1, CDK4, Myc, E-cadherin, N-cadherin and p-Rb in HT29 and RKO cells treated with si-ANKRD54 (K) or ELOVL6-IN-3 (L), Vemurafenib and combination. **M**,** N** Representative HE and IHC staining images of ANKRD54 (M) or ELOVL6 (N), Ki-67, CyclinD1 and p-ERK in xenograft tumor model of HT29 treated with shANKRD54 vector(M) or ELOVL6-IN-3 (N), Vemurafenib and combination. Scale bars of HE = 500 μm, Scale bars of magnification and IHC staining images = 25 μm. IHC staining quantitative analysis of Ki-67, CyclinD1 and p-ERK measured using ImageJ (**P < 0.01, ***P < 0.001). Data information: For (C, D, G-J), statistical significance was determined by the two-tailed Student’s t-test. For (M, N), statistical significance was determined by the nonparametric Mann-Whitney test

We next evaluated the therapeutic potential of targeting ANKRD54 or ELOVL6, particularly in combination with the BRAF^V600E^ inhibitor Vemurafenib (PLX4032). Xenograft models showed that either ANKRD54 inhibition or Vemurafenib treatment suppressed tumor growth, as reflected by reduced tumor volume, size, and weight, but combining the two produced a markedly superior therapeutic outcome compared to either monotherapy (Fig. 7A-D).

We then introduced ELOVL6‑IN‑3, a highly selective small‑molecule inhibitor of ELOVL6, into xenograft models (Fig. S7A), and first assessed its pharmacological effect using organoid viability assays. ELOVL6‑IN‑3 showed markedly lower IC50 values in BRAF‑MT PDOs and mouse organoids (11.67 µM and 12.42 µM) than in their BRAF‑WT counterparts (24.54 µM and 27.17 µM) (Fig. S7B, C), indicating a preferential dependency of BRAF‑MT models on ELOVL6 activity. Given that the ELOVL family comprises seven members with distinct substrate specificities, we evaluated the selectivity of ELOVL6‑IN‑3 and found that it selectively suppressed ELOVL6 mRNA and protein in HT29, RKO, and HCT116 cells, with only minor effects on ELOVL5 and ELOVL7 mRNA (Fig. S7D-G). In the PDO models, ELOVL6‑IN‑3 consistently suppressed ELOVL6 expression in a dose-dependent manner regardless of BRAF status. Correspondingly, reduced proliferation was only observed in BRAF-MT PDOs (Fig. S7H). In vivo toxicity was assessed by monitoring mouse body weight and endpoint hematoxylin & eosin (H&E) staining of multiple organs (lung, stomach, heart, liver, spleen, kidney), and no evidence of drug‑induced injury was observed (Fig. S7I, J). Xenograft experiments confirmed that ELOVL6‑IN‑3 alone inhibited BRAF‑MT CRC growth, and its combination with Vemurafenib led to a significantly enhanced therapeutic effect (Fig. 7E-H). This in vivo finding was consistently recapitulated in both BRAF‑MT PDOs and organoids from cKI mice (Fig. 7I, J).

At the molecular level, combining inhibition of ANKRD54 or ELOVL6 with Vemurafenib potently suppressed proliferative, metastatic, and MAPK/ERK signaling pathways in BRAF‑MT CRC cells (Fig. 7K, L). IHC analysis of xenograft tissues further validated that while each monotherapy moderately downregulated proliferation markers (Ki‑67, cyclin D1) and pERK, the combination therapy induced a significantly stronger downregulation (Fig. 7M, N). Thus, inhibiting the ANKRD54/ELOVL6 axis enhances the anti‑tumor activity of Vemurafenib.

### Association of the KAT2A/ANKRD54/CEBPB/ELOVL6 axis in BRAF-MT CRC

**Fig. 8:**
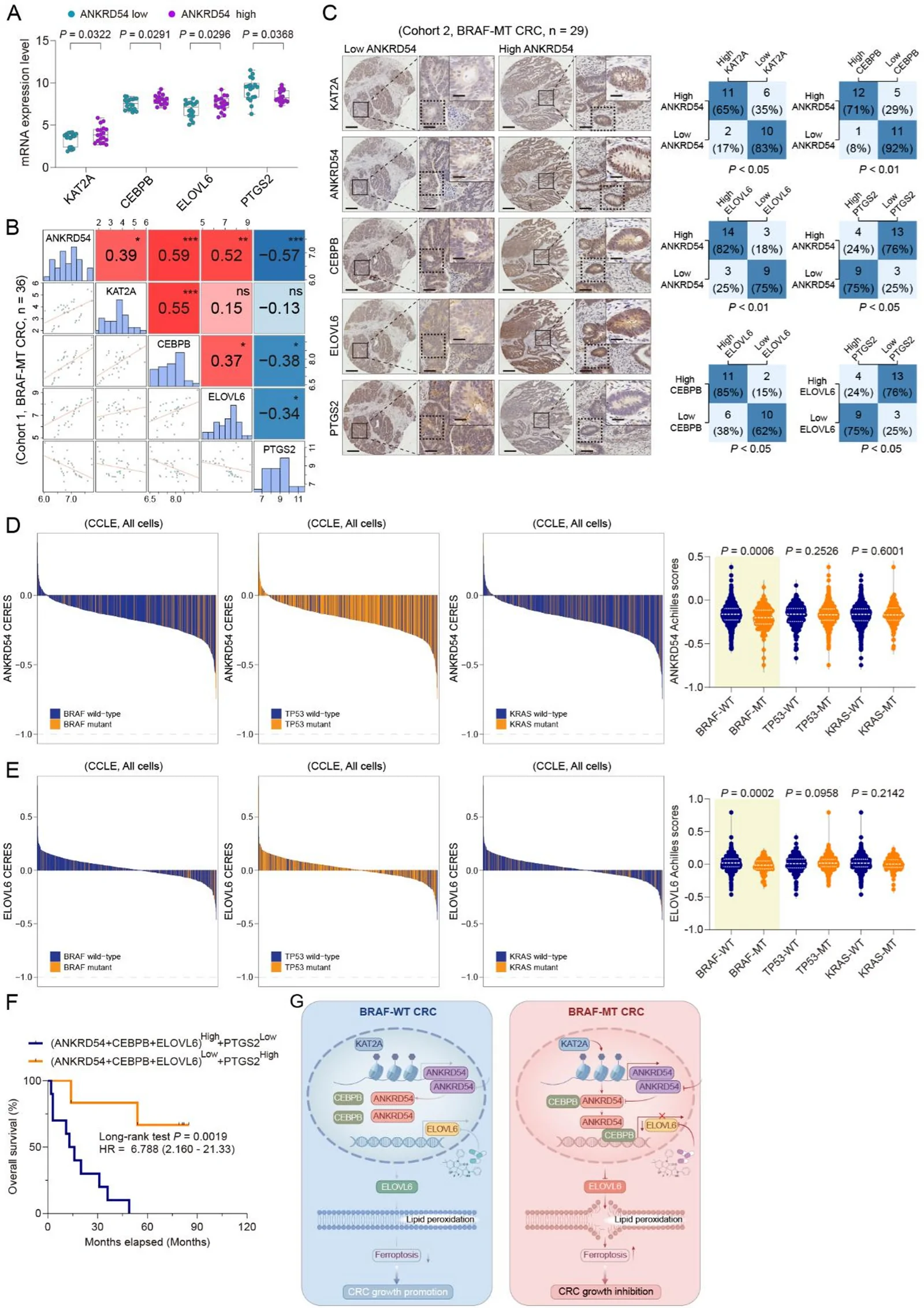
Association of the KAT2A/ANKRD54/CEBPB/ELOVL6 axis in BRAF-MT CRC. **A** qRT-PCR determination of KAT2A, CEBPB, ELOVL6 and PTGS2 mRNA levels of ANKRD54-low expression and ANKRD54-high expression BRAF MT CRC patients in Cohort 1 (*n* = 36; KAT2A: *P* = 0.0322; CEBPB: *P* = 0.0291; ELOVL6: *P* = 0.0296; PTGS2: *P* = 0.0368). **B** Correlation analysis of ANKRD54, KAT2A, CEBPB, ELOVL6, and PTGS2 in BRAF MT CRC of Cohort 1 (*n* = 36, *P < 0.05, **P < 0.01, ***P < 0.001, ns = no significance). **C** Representative IHC images and statistical analysis of ANKRD54, KAT2A, CEBPB, ELOVL6, and PTGS2 in BRAF MT CRC tissues with higher or lower ANKRD54 expression samples from Cohort 2 (*n* = 29). Right panels show enlarged images of indicated BRAF MT CRC tissues. Scale bars = 100 μm (left) and 10 μm (right), 1 μm (corner). **D** CERES dependency score for ANKRD54 across CCLE cell lines with BRAF status (*P* = 0.0006), TP53 status (*P* = 0.2526) and KRAS status (*P* = 0.6001). **E** CERES dependency score for ELOVL6 across CCLE cell lines with BRAF status (*P* = 0.0002), TP53 status (*P* = 0.0958) and KRAS status (*P* = 0.2142). **F** Kaplan–Meier survival curves of OS in BRAF MT CRC patients from Cohort 2 based on combined expression levels of ANKRD54, CEBPB, ELOVL6 and PTGS2 (*n* = 29, *P* = 0.0019). **G** Schematic diagram of the relationship among KAT2A, ANKRD54, CEBPB, ELOVL6, FA metabolism, ferroptosis and CRC progression. Data information: For (A, D, E), statistical significance was determined by the two-tailed Student’s t-test. For (B), statistical significance was determined by analysis of the Pearson correlation coefficient. For (C), statistical significance was determined by the Fisher exact test. For (F), statistical significance was determined by the log-rank test

We further validated the clinical relevance and ferroptosis‑suppressive role of the KAT2A‑mediated ANKRD54/CEBPB/ELOVL6 axis in BRAF‑MT CRC patient cohorts. In ANKRD54‑high BRAF‑MT tumors from Cohort 3, expression of KAT2A, CEBPB, and ELOVL6 was markedly elevated, while PTGS2 was reduced (Fig. 8A). This coordinated pattern was absent in BRAF‑WT CRC (Fig. S8A). Correlation analysis confirmed strong positive associations between ANKRD54 and its upstream regulator KAT2A, as well as its downstream effectors CEBPB and ELOVL6, and CEBPB and ELOVL6 were also positively correlated with each other. Conversely, PTGS2 expression was negatively correlated with ANKRD54, CEBPB, and ELOVL6 (Fig. 8B, S8B). These correlations were further corroborated at the protein level by IHC (Fig. 8C, S8C).

Moreover, DepMap CERES scores confirmed that BRAF-MT cells were more dependent on ANKRD54 and ELOVL6 than BRAF-WT cells (Fig. 8D, E), a pattern also observed across the broader panel of CRC cell lines (Fig. S8D). In contrast, cells harboring other common mutations (TP53, KRAS, NRAS, APC, PIK3CA) showed no such survival dependence (Fig. 8D, E, S8D-F). Survival analysis showed BRAF-MT CRC patients with high ANKRD54/CEBPB/ELOVL6 and low PTGS2 had poorer prognosis and shorter survival (Fig. 8F).

In summary, our findings delineate a previously unrecognized KAT2A/ANKRD54/CEBPB/ELOVL6 axis regulating FA metabolism and suppressing ferroptosis in BRAF-MT CRC, representing a synergistic therapeutic target to counteract malignancy and poor prognosis (Fig. 8G).

## Discussion

BRAF-MT CRC is characterized by high aggressiveness and therapeutic resistance, necessitating novel targeted strategies. We identified a novel ANKRD54-orchestrated metabolism-ferroptosis axis driving BRAF-MT CRC’s aggressive phenotype: KAT2A-mediated H3K27 acetylation enhances ANKRD54 transcription, ANKRD54 forms a complex with CEBPB to activate ELOVL6, and this cascade promotes malignancy via regulating FA metabolism and suppressing ferroptosis.

BRAF mutations result in the constitutive activation of the BRAF protein, which stimulates the downstream MAPK pathway via MEK to drive uncontrolled cell proliferation [35]. However, the efficacy of BRAF inhibitor monotherapy is often limited by acquired resistance [36]. ERK-mediated negative feedback relief induces RAS hyperactivation and BRAF-independent MEK reactivation [37]. Alternatively, resistance can arise through activation of parallel bypass pathways, such as PI3K/AKT signaling or upregulation of receptor tyrosine kinases (RTKs), which sustain pro‑proliferative [35, 38]. Therefore, combining BRAF and MEK inhibitors represents the standard approach to overcome resistance, underscoring the need for further development of effective MEK‑targeting agents.

Our study revealed that ANKRD54 may act as a novel upstream regulator of the MAPK pathway, as its targeted inhibition significantly attenuated MEK and ERK phosphorylation. BRAF-MT CRC exhibits intrinsic resistance to MAPK pathway inhibitors (MAPKi), with only ~ 20% objective response rate (ORR) in the BEACON trial [39]. Beyond canonical MAPK reactivation, additional resistance mechanisms have been reported in BRAF-MT tumors, as BRAFi ± EGFRi were shown to activate the PLK1/CBX8/GPX4 axis in a MAPK-independent manner, upregulating GPX4 to counteract ferroptosis [40]. Our study identified high ANKRD54 expression in BRAF‑MT CRC is independent of the BRAF mutation itself, but is epigenetically driven by KAT2A via H3K27ac at the ANKRD54 promoter. ANKRD54 further regulates ELOVL6 expression, and this ANKRD54/ELOVL6 axis complements the network described above through FA reprogramming, providing a mechanistic explanation for the intrinsic MAPKi resistance in BRAF-MT CRC. CCLE database and our patient cohort data first suggested differential activation of this network, which we subsequently confirmed functionally in animal models and PDOs, supporting the synergistic potential of combining ANKRD54 inhibition with BRAFi. Besides, KAT2A typically acetylates multiple histone sites (e.g., H3K9, H3K14) [41], and has been reported to promote ferroptosis in diabetic cardiomyopathy and enhance proliferation and metastasis in nasopharyngeal carcinoma through H3K27ac [42, 43]. It can also stabilize oncoproteins such as MYCN via non‑histone acetylation [44]. In CRC, BRAF mutations were detected in approximately 28% of MSI cases, compared with only 7.5% of microsatellite-stable (MSS) cases [45]. The MSI-H CRC, which exhibits higher mutation rates, is considered more genetically driven [46]. Notably, in the large MSS subgroup, KAT2A inhibition has been shown to suppress tumor growth [47]. Thus, our findings suggest that KAT2A‑mediated upregulation of ANKRD54 sustains MAPK signaling in BRAF‑MT CRC, and co‑inhibition of KAT2A/ANKRD54 with BRAF represents a rational therapeutic strategy.

While ankyrin repeat-containing proteins of the ANKRD family are known to mediate protein-protein interactions [14, 48], their specific roles in CRC have remained unclear. Here, we demonstrate that ANKRD54 functions as a transcriptional co‑regulator by binding to the transcription factor CEBPB. CEBPB is a well‑established regulator of FA metabolism whose activity depends on the ratio between its activating isoforms (LAP*/LAP) and the inhibitory isoform (LIP) [49], enabling context‑dependent functions. For example, CEBPB promotes FA oxidation to support stemness and chemoresistance in gastric cancer [50], whereas in ulcerative colitis it exacerbates ferroptosis by regulating downstream gene expression [51]. Our results show that the ANKRD54‑CEBPB complex activates transcription of ELOVL6, a central enzyme in FA elongation, thereby regulating FA metabolism in BRAF‑MT CRC.

BRAF‑MT cells display imbalanced saturated/monounsaturated FA ratios and accumulate specific long‑chain FAs (C18-C22) [52]. Fatty acid elongation, orchestrated by the ELOVL enzyme family, likely plays a vital role in FA metabolism and ferroptosis regulation in BRAF‑MT CRC. The ELOVL family exerts dual effects on ferroptosis sensitivity, which is determined by the dynamic balance between pro‑ferroptotic polyunsaturated fatty acids (PUFAs) and anti‑ferroptotic monounsaturated fatty acids (MUFAs) in cell membranes. For instance, ELOVL7 induces ferroptosis in podocytes through accumulation of long‑chain PUFA‑containing lipids [53], while ELOVL5 exacerbates ferroptosis in diabetic nephropathy by increasing synthesis of PUFAs such as arachidonic acid [54]. In contrast, ELOVL6 antagonizes ferroptosis. Mechanistically, it catalyzes the elongation of C16 to C18 FAs, providing substrate for oleic acid (C18:1) synthesis the most abundant MUFA in mammalian cells [55]. These stable MUFAs compete with PUFAs for incorporation into membrane phospholipids, thereby diluting the pool of oxidizable substrates and establishing a protective barrier against lipid peroxidation. Beyond this role, ELOVL6 has multifaceted pathological functions. It synergizes with ceramide synthase 4 (CerS4) to produce ceramides that induce insulin resistance via Akt inhibition, making it a potential target for metabolic disorders [56]. In cancer, targeting ELOVL6 enhances chemosensitivity in pancreatic cancer by disrupting c-MYC-driven FA metabolism [57] and can induce degradation of oncogenic KRAS‑G12V through modulation of membrane lipid composition [58]. Building on these insights, we identified that the KAT2A‑mediated ANKRD54/CEBPB/ELOVL6 axis regulates FA metabolism and shields tumor cells from ferroptosis. We further demonstrated that ELOVL6-IN-3 [59], a novel ELOVL6 inhibitor, increases intracellular oxidative stress and lipid peroxidation, leading to ferroptosis and inhibition of tumor growth. Combining Vemurafenib with ANKRD54/ELOVL6 axis inhibition exhibited superior efficacy compared to monotherapy in BRAF‑MT CRC models, highlighting a promising therapeutic strategy. Translational feasibility of ANKRD54 targeting merits consideration. Its selective dependency in BRAF-MT CRC suggests potential for this patient population. Preclinical siRNA efficacy could be advanced via small-molecule inhibitors, peptide agents, and nanoparticle delivery systems, while ELOVL6-IN-3 offers a downstream option. Combination with BRAFi may warrant further preclinical optimization for safety and efficacy in ferroptosis-driven resistance. However, this study has several limitations. The number of BRAF-MT CRC patients in our cohort was relatively small, and the association between ANKRD54 expression and prognosis was analyzed retrospectively. Therefore, the prognostic value of ANKRD54 in BRAF-MT CRC requires further validation in larger, multicenter prospective cohorts. Despite the inclusion of three clinical cohorts, larger independent datasets and prospective validation are still needed to fully establish its clinical utility, and we consider this an important future direction.

In conclusion, our study delineates a complete, epigenetically driven signaling cascade (KAT2A-mediated histone acetylation to ANKRD54/CEBPB-dependent ELOVL6 activation) underlying BRAF‑MT CRC. The enhanced efficacy of BRAF inhibitor combined with this metabolic axis inhibition supports this therapeutic approach, offering potential to improve BRAF-MT CRC patient outcomes.

## Materials and methods

### Cell culture and treatments

Human CRC cell lines (LOVO, HT29, COLO201, COLO205, RKO, SW480, SW1116 and HCT116) from the American Type Culture Collection (ATCC) were cultured in recommended media containing 10% FBS (ExCell Bio, Shanghai, China) and 1% penicillin-streptomycin at 37 °C with 5% CO_2_. Gene knockdown was achieved by transfecting cells with target or control siRNAs (Genechem, Shanghai, China) using Lipofectamine 3000 (Thermo Fisher Scientific, Waltham, MA, USA), with efficiency confirmed by qRT-PCR/western blot at 48–72 h post-transfection. Stable cell lines were established through lentiviral transduction (Genechem, Shanghai, China) in the presence of 5 µg/mL Polybrene and subsequent puromycin selection. Refer to Supplementary Table 1 for all siRNA sequences used.

### Patient specimens

This study analyzed three distinct cohorts of surgically treated CRC patients from 2014 to 2020. Cohort 1 consisted of 158 paired CRC and adjacent normal tissues. Cohorts 2 and 3 comprised 104 and 158 patients, respectively, with detailed clinicopathological data available for Cohort 2. All CRC diagnoses were confirmed by histopathology. For subsequent biomolecular analyses, tissues were either snap-frozen in liquid nitrogen for RNA extraction or embedded in paraffin. Patients who had received any preoperative anticancer therapy were excluded. The study adhered to the ethical standards of the 1975 Declaration of Helsinki and received approval from the Ethics Committee of the Ninth People’s Hospital, Shanghai Jiao Tong University School of Medicine, with informed consent obtained from all participants. Clinical details for Cohort 2 are provided in Supplementary Table 2.

### CRC Xenograft Model

Four-week-old male BALB/c nude mice, sourced from Charles River Laboratories in Beijing, were maintained in a specific pathogen-free (SPF) setting within the IVC facility at Shanghai Jiao Tong University, with unrestricted access to food and water. For the establishment of a CRC xenograft model, tumor cells were injected subcutaneously into the right side of each mouse. Once tumors were palpable, tumor size was measured every 2 or 3 days to track tumor growth. The tumor volume was determined using the formula: Volume = (length × width²) × 0.5. The mice received oral gavage of either Vemurafenib (at a dose of 40 mg/kg, Roche, Basel, Switzerland) or ELOVL6-IN-3 (at a dose of 30 mg/kg, MCE, Monmouth Junction, NJ, USA) on alternate days. After 20 or 28 days of treatment, the mice were euthanized to collect and weigh the subcutaneous tumors. All animal experiments adhered to the guidelines outlined in the National Institutes of Health Guide for the Care and Use of Laboratory Animals and received approval from the Animal Ethics Committee of Shanghai Jiao Tong University.

### Organoid culture and staining

PDOs and mouse intestinal organoids were established from fresh tissues. Details of Vil-CRE; Braf^LSL−V637E/+^ mice model construction are provided in previous study by Allan B. etc. [60]. Minced tissues were washed in ice-cold PBS supplemented with penicillin/streptomycin (#15140122, Gibco, Thermo Fisher Scientific, Waltham, MA, USA). The tissue was then dissociated at 37 °C for 30 min in a digestion buffer containing 500 U/mL collagenase IV (#C9407, Sigma-Aldrich, St. Louis, MO, USA), 1.5 mg/mL collagenase II (#17101015, Gibco, Thermo Fisher Scientific, Waltham, MA, USA), 20 mg/mL hyaluronidase (#H3884, Sigma-Aldrich, St. Louis, MO, USA), 0.1 mg/mL dispase type II (#D4693, Sigma-Aldrich, St. Louis, MO, USA), and 10 µM Y-27,632 (Wako, Japan, 036-24023). Following filtration and gravity sedimentation, the crypt-containing fraction was collected, centrifuged (300 g, 3 min, 4 °C), and the resulting pellet was resuspended in Matrigel (#356231, Corning, Corning, NY, USA) for plating.

The CRC organoid culture medium consisted of Advanced DMEM/F12 (#12634028, Gibco, Thermo Fisher Scientific, Waltham, MA, USA) supplemented with 10 mM HEPES (#15630080, Gibco, Thermo Fisher Scientific, Waltham, MA, USA), 2mM GlutaMAX (#35050061, Gibco, Thermo Fisher Scientific, Waltham, MA, USA), 1× Primocin (#ant-pm-05, InvivoGen, San Diego, CA, USA), 1× B-27 Supplement (#17504044, Gibco, Thermo Fisher Scientific, Waltham, MA, USA), 1mM N-acetyl-L-cysteine (#A9165, Sigma-Aldrich, St. Louis, MO, USA), 50 ng/mL EGF (#PMG8041, Gibco, Thermo Fisher Scientific, Waltham, MA, USA), 100 ng/mL Noggin (#250 − 38, PeproTech, Rocky Hill, NJ, USA), 10% R-spondin 1 conditioned medium, 10 nM gastrin I (#3006, Tocris, Bristol, UK), 10 mM Nicotinamide (#2939, Tocris, Bristol, UK), 100 ng/mL IGF-I (#590908, BioLegend, San Diego, CA, USA), 50 ng/mL FGF-basic (#100-18B, PeproTech, Rocky Hill, NJ, USA ), and 10 nM Prostaglandin E2 (#P6532, Sigma-Aldrich, St. Louis, MO, USA). The medium was replaced every 2–3 days. For subculturing, organoids were dissociated with TrypLE Express Enzyme (#12605028, Gibco, Thermo Fisher Scientific, Waltham, MA, USA), and 10 µM Y-27,632 was added to the medium for the first two days to prevent anoikis.

For immunofluorescence staining, organoids were fixed in 4% fresh formaldehyde for 30 min at room temperature, followed by sectioning, deparaffinization, and rehydration. After permeabilization and blocking, sections were incubated overnight at 4 °C on a rotator with the following primary antibodies: Ki-67 (#AB9260, Sigma-Aldrich, St. Louis, MO, USA), EpCAM/TROP-1 (#AF960, R&D Systems, Minneapolis, MN, USA), ANKRD54 (#25738-1-AP, Proteintech, Rosemont, IL, USA), C/EBP beta (#PA5-27244, Invitrogen, Thermo Fisher Scientific, Waltham, MA, USA), 4-Hydroxynonenal (#bs-6313R, Bioss, Beijing, China), PTGS2 (#ab179800, Abcam, Cambridge, MA, USA), and ELOVL6 (#ab69857, Abcam, Cambridge, MA, USA). The next day, sections were incubated with corresponding secondary antibodies for 3 h at room temperature in the dark. Finally, stained organoids were mounted on slides using DAPI Fluoromount-G (#ENZ-52404, Enzo Life Sciences, Farmingdale, NY, USA), and images were acquired with a confocal microscope.

### Quantitative RT-PCR analysis (qRT-PCR)

Total RNA was isolated utilizing TRIzol reagent (Ambion, Thermo Fisher Scientific, Austin, TX, USA). The resulting RNA was purified via treatment with Deoxyribonuclease I (Sigma-Aldrich, St. Louis, MO, USA) and a subsequent phenol-chloroform extraction. Messenger RNA was then reverse-transcribed into cDNA using the PrimeScript RT Reagent Kit (TAKARA, Kusatsu, Shiga, Japan). Quantitative real-time PCR (qPCR) reactions were conducted in triplicate on a StepOnePlus system (Applied Biosystems, Thermo Fisher Scientific, MA, USA). Gene expression fold change was calculated using the comparative CT (−ΔΔCt) method. All primer sequences are available in Supplementary Table 1.

### Western blot analysis

Protein lysates were prepared by lysis in RIPA buffer (Beyotime Biotechnology, Shanghai, China) with continuous agitation for 60 min on ice. After centrifugation at 12,000 × g for 20 min at 4 °C, the supernatant was collected, and protein concentration was quantified using a BCA Assay Kit (Thermo Fisher Scientific, MA, USA). Equal amounts of protein were separated by electrophoresis on 10% SDS-polyacrylamide gels and subsequently transferred onto PVDF membranes (Bio-Rad Laboratories, Hercules, CA, USA). The membranes were blocked for 2 h with 5% BSA, followed by overnight incubation at 4 °C with specific primary antibodies. After washing with TBST, membranes were incubated with HRP-conjugated secondary antibodies (Kangcheng, Shanghai, China). Protein bands were visualized using an ECL detection system (Bio-Rad Laboratories, Hercules, CA, USA). The following primary antibodies were utilized: ANKRD54 (#25738-1-AP), N-cadherin (#22018-1-AP), SLC7A11 (#26864-1-AP), ACLY (#15421-1-AP), ACC1 (#21923-1-AP), FADS2 (#28034-1-AP), ELOVL6 (#21160-1-AP) and ACSL3 (#20710-1-AP) were acquired from Proteintech (Wuhan, China). ACSL4 (#sc-365230), FTH1 (#sc-376594), FDFT1 (#sc-271602), HMOX1 (#sc-136960), ACOX1 (#sc-517306), ACOT1/2 (#sc-373917), FASN (#sc-55580), PLA2G (#sc-376563), ERK1/2 (#sc-514302), Myc (#sc-40), ACSL5 (#sc-365478), SCD (#sc-58420), p-RB (#sc-377539), MEK-1 (#sc-6250), p-MEK (#sc-81503), pERK (#sc-7383), HSPB1 (#sc-13132) and PTGS2 (#sc-19999) were sourced from Santa Cruz Biotechnology (Santa Cruz, CA, USA). TUBULIN (#ab176560), H3 (#AB1791) and H3K27ac (#AB4729) were obtained from Abcam (Cambridge, MA, USA). KAT2A (#3305T) and E-cadherin (#3195T) were sourced from CST (Danvers, MA, USA). CyclinD1 (#SDT-R423) and CDK4 (#S-685-210) were purchased from STARTER (SDT, Shanghai, China).

### Cell proliferation assessment

Cell viability was evaluated using Cell Counting Kit-8 (Dojindo, Kumamoto, Japan). Stable transfected and control CRC cells were plated in 96-well plates (1–3 × 10^3^cells/well) and cultured for 4 days. CCK8 reagent (10 µL/well) diluted in serum-free medium was added daily, followed by 2-hour incubation at 37 °C in darkness. Absorbance was measured at 450 nm. For colony formation assays, cells were seeded in 6-well plates and cultured for 7–10 days. Colonies were fixed with 4% paraformaldehyde, stained with 0.1% crystal violet, and counted (clusters > 50 cells defined as colonies).

### Organoid viability assay

The determination of IC50 values was performed as previously described [61]. Briefly, a series of 3-fold serial dilutions of ELOVL6-IN-3 (0–90 µM) was prepared in complete organoid culture medium based on Advanced DMEM/F12. CRC-PDOs and mouse organoids were treated with the indicated concentrations, and organoid viability was measured using an ATP-based luminescence assay. IC50 values were derived from dose–response curves. Three independent experiments in duplicate were performed.

### Cell invasion and migration assays

Transwell chambers (Corning, Corning, NY, USA) were used to assess cell invasion and migration. For invasion assays, membranes were precoated with Matrigel (BD Biosciences, San Jose, CA, USA), whereas uncoated membranes were applied for migration assays. CRC cells (5–50 × 10⁴ /well) suspended in serum-free medium were seeded into the upper chambers, and medium containing 10% fetal bovine serum (FBS) was added to the lower chambers as a chemoattractant. After incubation at 37 °C for 24 h (migration) or 48 h (invasion), cells adhering to the bottom of the membranes were fixed, stained with crystal violet, and counted in five random fields per well. All experiments were performed in triplicate and repeated three times independently.

### Immunohistochemical (IHC) & H&E staining

Prior to IHC staining, tissue sections were subjected to H&E staining for pathological observation (hematoxylin staining for 5 min, eosin staining for 30 s, followed by dehydration, clearing, and mounting). For IHC analysis, antigen retrieval was performed in citrate buffer using microwave heating. Endogenous peroxidase activity was blocked with 3% H₂O₂, and sections were then blocked with 5% normal goat serum. Primary antibodies were incubated with sections overnight at 4 °C in a humidified chamber. Horseradish peroxidase (HRP)-conjugated secondary antibodies and 3,3’-diaminobenzidine (DAB) substrate were used for signal detection, followed by hematoxylin counterstaining.

Staining evaluation was conducted based on two parameters: staining intensity (0, negative; 1, weak; 2, moderate; 3, strong) and positive cell percentage (0, < 5%; 1, 5–25%; 2, 25–50%; 3, 50–75%; 4, > 75%). The final immunoreactive score (IRS) was calculated as the product of the two parameters (range: 0–12), and classified as negative (0), low (1–4), medium (6–8), or high (9–12) expression. Protein expression in mouse xenografts and clinical CRC specimens was quantified using Image-Pro Plus 6.0 (Media Cybernetics, Rockville, MD, USA) based on staining intensity and scope. The antibodies used were: ANKRD54 (#25738-1-AP), ELOVL6 (#21160-1-AP), PTGS2 (#sc-19999), CyclinD1 (#SDT-R423), pERK (#sc-7383), KAT2A (#3305T), Ki-67 (#AB9260, Sigma-Aldrich, St. Louis, MO, USA), 4-HNE (#MA5-27570, Invitrogen, Thermo Fisher Scientific, Waltham, MA, USA), Cleaved Caspase-3 (#9661, Cell Signaling Technology, Danvers, MA, USA) and CEBPB (#sc-7962).

### Co-immunoprecipitation (Co-IP)

Cells were lysed in immunoprecipitation (IP) buffer supplemented with protease/phosphatase inhibitors (Beyotime Biotechnology, Shanghai, China). Cleared cell lysates were incubated with specific primary antibodies or control immunoglobulin G (IgG) overnight at 4 °C, followed by incubation with Protein A/G Beads (Santa Cruz Biotechnology, Dallas, TX, USA) for 3 h. Precipitated proteins were analyzed by western blotting. Antibodies used included: ANKRD54 (#25738-1-AP), CEBPB (#sc-7962), H3K27ac (#ab4729), and KAT2A (#3305T).

### Chromatin immunoprecipitation (ChIP)

ChIP assays were performed using the EZ-Magna ChIP Kit (Millipore, Burlington, MA, USA) following the manufacturer’s protocol. After formaldehyde cross-linking, cells were lysed and sonicated to generate chromatin fragments. Target chromatin was immunoprecipitated with specific antibodies or control IgG. The precipitated DNA was analyzed by PCR using primers listed in Supplementary Table 1. Antibodies used were: ANKRD54 (#25738-1-AP), CEBPB (#sc-7962), H3K27ac (#ab4729), and KAT2A (#3305T).

### Oil red O staining

Frozen xenograft Sect.  (10 μm) were stained with Oil Red O for 12 min, followed by hematoxylin counterstaining, differentiation in hydrochloric acid-ethanol for 2–3 s, bluing in water for 3 min, ammonia treatment for 3–4 s to enhance bluing, and mounting with glycerol. Images were captured using a digital trinocular microscope at 400× magnification in three randomly selected regions. The area of lipid droplets was quantified using ImageJ software (NIH), and the percentage of lipid droplet area was calculated as (lipid droplet area / total field area) × 100%.

### FFA detection and TG analysis

Transfected CRC cells were seeded into 6-well plates and treated with chemicals for 48 h. Cells or tissues were lysed with RIPA buffer for 25 min. FFA and TG levels were detected using the FFA Detection Kit (#ab65341, Abcam, Cambridge, MA, USA) and TG Assay Kit (#ab65336, Abcam, Cambridge, MA, USA), respectively. Protein concentration was measured using the BCA Protein Assay Kit (Thermo Fisher Scientific, Waltham, MA, USA) for data normalization.

### Oxidative stress-induced lipid peroxidation assay

CRC cells were trypsinized, pelleted, and washed with PBS before lysis. Intracellular GSH levels were measured using the GSH Kit (#DIGT-250, BioAssay Systems, Hayward, CA, USA) according to the manufacturer’s instructions, with detection at 412 nm using a microplate reader. Intracellular ROS levels were detected using the fluorescent probe 2’,7’-dichlorodihydrofluorescein diacetate (DCFH-DA) (#D6883, Sigma-Aldrich, St. Louis, MO, USA) followed by flow cytometry and laser scanning confocal microscopy. MDA content was determined using the MDA Assay Kit (A003-1, Jiancheng Bioengineering, Nanjing, China), with detection at 532 nm. Lipid peroxidation was assessed using the BODIPY 581/591 C11 probe (#D3861, Invitrogen, Thermo Fisher Scientific, Waltham, MA, USA). Organoids were incubated with 10 µM probe at 37 °C for 1 h in the dark. The green/red fluorescence intensity ratio was quantified using ImageJ software, and representative images were obtained by channel merging and contrast adjustment.

### Luciferase assay

Luciferase activity was measured using the Dual Luciferase Reporter Assay System (Promega, Madison, WI, USA). CRC cells seeded in 96-well plates were co-transfected with reporter plasmids (PGL3-based vectors containing wild-type or mutant 3’-untranslated region (UTR) sequences) and effector molecules (siRNAs). After 48 h of transfection, firefly and Renilla luciferase activities were measured sequentially, and normalized luciferase activity was calculated as the firefly/Renilla ratio.

### Statistical methods

Data were analyzed using SPSS 24.0 (IBM) and GraphPad Prism 8.0. All experiments were performed at least three times independently, and data are presented as mean ± standard deviation (SD). Student’s t-test was used for comparison of normally distributed continuous data between two groups, while the Mann-Whitney U test was used for non-normally distributed data. Spearman’s correlation analysis was used to assess correlations between variables. Categorical data were analyzed using the Chi-square test or Fisher’s exact test. Survival analysis was performed using the Kaplan-Meier method with the log-rank test. A *P*-value < 0.05 was considered statistically significant.

## Supplementary Information

Supplementary Material 1.

Supplementary Material 2.

Supplementary Material 3.

## Data Availability

All genomic data from the CCLE are available at https://portals.broadinstitute.org/ccle/data. DepMap 21Q4 was used for all analyses. The other data are publicly available in the NCI Genomic Data Commons (GDC) portal at https://portal.gdc.cancer.gov/, and were generated by The Cancer Genome Atlas (TCGA) Research Network. Other high-throughput sequencing data can be downloaded from NCBI GEO database under the accession numbers GSE103479 (https://www.ncbi.nlm.nih.gov/geo/query/acc.cgi?acc=GSE103479),GSE13067 (https://www.ncbi.nlm.nih.gov/geo/query/acc.cgi?acc=GSE13067),GSE39582 (https://www.ncbi.nlm.nih.gov/geo/query/acc.cgi?acc=GSE39582), GSE19234 (https://www.ncbi.nlm.nih.gov/geo/query/acc.cgi?acc=GSE19234), GSE15605 (https://www.ncbi.nlm.nih.gov/geo/query/acc.cgi?acc=GSE15605) and GSE39084 (https://www.ncbi.nlm.nih.gov/geo/query/acc.cgi?acc=GSE39084).

## Abbreviations

MT: Mutant
CRC: Colorectal cancer
ANKRD54: Ankyrin repeat domain 54
PDOs: Patient-derived organoids
FA: Fatty acid
CEBPB: CCAAT/enhancer-binding protein beta
KAT2A: Lysine acetyltransferase 2 A
ACSL3: Acyl-CoA synthetase long-chain family member 3
CIMP: CpG island methylator phenotype
MSI: Microsatellite instability
Dmmr: Mismatch repair deficiency
GPX4: Glutathione peroxidase 4
GSH: Glutathione
PTGS2: Prostaglandin-endoperoxide synthase 2
SLC7A11: Solute carrier family 7 member 11
ROS: Reactive oxygen species
H3K27ac: H3K27 acetylation
ChIP: Chromatin immunoprecipitation
IHC: Immunohistochemistry
GSEA: Gene set enrichment analysis
TG: Triglyceride
FFA: Free Fatty acid
ELOVL6/: ELOVL fatty acid elongase 6
MDA: Malondialdehyde
CPT1: carnitine palmitoyl transferase 1
SLC6A14: solute carrier family 6 member A14
PUFAs: Polyunsaturated fatty acids
MUFAs: Monounsaturated fatty acids
SPF: Specific pathogen-free
Co-IP: Co-Immunoprecipitation
