# Supplementary material for: Targeting the ANKRD54/ELOVL6 axis suppresses malignancy in BRAF mutant colorectal cancer by disrupting fatty acid metabolism and promoting ferroptosis
Source: J Exp Clin Cancer Res. 2026 Aug 11;45:231. doi: 10.1186/s13046-026-03809-0 (PMC13741422; doi:10.1186/s13046-026-03809-0)
Supplement: Supplementary file 1 [file 13046_2026_3809_MOESM3_ESM.docx]

**FIG S1**

**A** Expression analysis of ANKRD54 mRNA levels in BRAF MT versus BRAF WT CRC from GEO datasets (GSE39582, n=512, *P*=0.0007).

**B** Comparison of clinicopathological characteristics between ANKRD54 high- and low-expression tumors in BRAF WT CRC patients from Cohort 2 (n=70).

**C** Kaplan-Meier survival curves of OS (GSE19234, n=44, *P*=0.0103) or PFS (GSE19234, n=44, *P*=0.0103) in melanoma patients from GEO database based on expression levels of ANKRD54.

**D, E** Kaplan-Meier survival curves of OS and PFS in BRAF WT or BRAF MT CRC (BRAF WT: OS, *P*=0.0372, PFS, *P*=0.0414; BRAF MT: OS, *P*=0.1642, PFS, *P*=0.0033); (D) and melanoma (BRAF WT: OS, *P*=0.6766, PFS, *P*=0.7378; BRAF MT: OS, *P*=0.0405, PFS, *P*=0.0074) (E) patients from TCGA database based on ANKRD54 expression levels.

**F, G** Univariate Cox regression analyses of prognostic factors in BRAF-MT (F) and BRAF-WT (G) CRC of Cohort 2.

**H, I** Multivariate Cox regression analyses of prognostic factors in BRAF-MT (H) and BRAF-WT (I) CRC of Cohort 2.

Data information: For (A), statistical significance was determined by the two-tailed Student’s t-test. For (B), statistical significance was determined by the Chi-square test. For (C-E), statistical significance was determined by the log-rank test. For (F-I), statistical significance was determined by Cox proportional hazards regression model.

**FIG S2**

**A** Western blot analysis of ANKRD54, p-ERK, ERK, p-MEK, and MEK in RKO following transfection with the indicated V600 mutant forms of BRAF (V600D, V600E, V600K, V600L, V600M and V600R) vectors and BRAF WT vector.

**B, C** Correlation analysis of ANKRD54 expression with KAT2A, KAT5, KAT8, KAT14, HAT1, CREBBP, EP300, KAT7, KAT2B, KAT6A, and KAT6B in colon (B) and rectal (C) cancer from TCGA database.

**D** Western blot analysis of KAT2A, p-ERK, ERK, p-MEK and MEK in 293T cells with overexpression of WT-BRAF and BRAF mutants (V600D, V600E, V600K, V600L, V600M, and V600R).

**E** Western blot analysis of KAT2A, ANKRD54 and H3K27ac in BRAF WT cells (HCT116) transfected with control siRNA or KAT2A siRNAs.

**F-H** Western blot analysis of ANKRD54 and H3K27ac in BRAF WT cells (HCT116) treated with KAT2A inhibitors Butyrolactone 3 (E), PU139 (F), and CPTH2 (G) at indicated concentration.

Data information: For (B, C), statistical significance was determined by analysis of the Pearson correlation coefficient.

**FIG S3**

**A** CERES scores of all members of ANKRD family in BRAF MT vs BRAF WT CRC cell lines.

**B** Cell viability assay was performed in BRAF WT CRC cell lines (HCT116, SW480, SW1116, Lovo) transfected with si-NC or si-ANKRD54s. Data are presented as mean ± SD (biological replicates, n=5, ns= no significance).

**C, D** Transwell invasion and migration assays were performed and analyzed in BRAF MT CRC cell lines (COLO201, COLO205, HT29, RKO) and BRAF WT CRC (HCT116, SW480, SW1116) following ANKRD54 inhibition. Data are presented as mean ± SD (biological replicates, n= 3, ns= no significance, ****P<*0.001).

**E-G** Representative images of tumors and analysis in nude mice intervened with shNC or shANKRD54 BRAF WT (HCT116) cells. The stripped tumors are shown (E) and growth curve was plotted (F). The tumors were extracted and weighted after 20 days (G). Data are presented as mean ± SD (biological replicates, n=6).

Data information: For (A, B, D, F, G), statistical significance was determined by the two-tailed Student’s t-test.

**FIG S4**

**A-F** GSEA analyses of multiple tumorigenic gene sets correlated with ANKRD54 expression, including CRC signaling, cell cycle, ERK pathway, cell proliferation, fatty acid metabolism, cell metastasis.

**FIG S5**

**A** Representative confocal images showing the ANKRD54-knockdown PDOs (CRC-45 and CRC-87) was stably generated (Scale bar = 50 μm). ANKRD54 (Red) and DAPI (Blue). Luminescence of ANKRD54 staining was calculated and compared from 10 random fields per sample (CRC-45: *P*=0.0070; CRC-87: *P*=0.0104).

**B** Representative confocal images showing Ki-67, ELOVL6, EPCAM and DAPI staining in CRC-45 PDO transfected with control vector and shANKRD54 vector (Scale bar = 50 μm). Ki-67 (Green), ELOVL6 (Red), EPCAM (Pink) and DAPI (Blue). Luminescence of ELOVL6 staining was calculated and compared from 10 random fields per sample (*P*=0.0969).

**C** Association between ANKRD54 and 10 candidate genes of FA biosynthesis was analyzed by Pearson’s correlation analysis in cBioPortal CRC database (**P<*0.05, ***P<*0.01, ****P<*0.001, ns= no significance).

**D** Representative confocal images showing the CEBPB-knockdown PDOs (CRC-45 and CRC-87) was stably generated (Scale bar = 50 μm). CEBPB (Red) and DAPI (Blue). Luminescence of CEBPB staining was calculated and compared from 10 random fields per sample (CRC-45: *P=*0.0093; CRC-87: *P=*0.0014).

**E** Representative confocal images showing Ki-67, ELOVL6, EPCAM and DAPI staining in CRC-45 PDO transfected with control vector and shCEBPB vector (Scale bar = 50 μm). Ki-67 (Green), ELOVL6 (Red), EPCAM (Pink) and DAPI (Blue). Luminescence of ELOVL6 staining was calculated and compared from 10 random fields per sample (*P*=0.5716).

**F, G** Bright-field microscopy images of PDOs (CRC-87 and CRC-45) and organoid derived from C57BL/6 mice colorectal tissues and Vil-CRE;BRAF^LSL-V637E/+^ mice colorectal tissues with CEBPB knockdown. Organoid diameters were measured using ImageJ (Scale bar = 100 μm, ***P*<0.01, ****P*<0.001, ns= no significance).

**H** Luciferase activity in RKO cells with CEBPB knockdown following transfection with ELOVL6 promoter luciferase reporter vectors (biological replicates, n=4; ****P<*0.001, ns= no significance).

**I** Luciferase activity in RKO cells with ANKRD54 knockdown following transfection with CEBPB siRNA and ELOVL6 WT promoter luciferase reporter vectors (biological replicates, n=4; ****P<*0.001, ns= no significance).

**J, K** Western blot analysis of CEBPB and ANKRD54 in HT29 and RKO cells treated with ANKRD54 siRNAs and CHX at different time points.

**L** Representative confocal images showing CEBPB, ANKRD54 and DAPI staining in organoids transfected with control vector and shANKRD54 vector (Scale bar = 50 μm). ANKRD54 (Green), CEBPB (Red), and DAPI (Blue). Luminescence of CEBPB staining was calculated and compared from 10 random fields per sample (ns= no significance).

**M** Luciferase activity of CEBPB in HT29 and RKO cells with ANKRD54 overexpression (biological replicates, n=4; ****P<*0.001, ns= no significance).

**N** ChIP-qRT-PCR assays verified the potential ANKRD54-binding and CEBPB‐binding site in the ELOVL6 promoter region of RKO cells after inhibition of CEBPB and ANKRD54. IgG was used as a control (biological replicates, n=3; ****P*<0.001).

Data information: For (C), statistical significance was determined by analysis of the Pearson correlation coefficient. For (A, B, D, E, L), statistical significance was determined by the nonparametric Mann-Whitney test. For (F-K, M, N), statistical significance was determined by the two-tailed Student’s t-test.

**FIG S6**

**A, B** Cell viability assay following ANKRD54 knockdown in HT29, RKO and HCT116 cells was measured by trypan blue staining. Data are mean ± SD (biologically independent samples, n=3, ****P*<0.001, ns= no significance).

**C-H** The GSH levels in HT29, RKO and HCT116 cells following ANKRD54(C) or ELOVL6 (D) knockdown were measured by GSH assay kit. The MDA levels in HT29, RKO and HCT116 cells following ANKRD54(E) or ELOVL6 (F) knockdown were measured by TBA colorimetric assay. The ROS levels in HT29, RKO and HCT116 cells following ANKRD54(G) or ELOVL6 (H) knockdown were measured by flow cytometry analysis of CM-H2DCFDA staining. (biologically independent samples, n=3, **P*<0.5, ****P*<0.001, ns= no significance).

**I** Representative confocal images showing ROS, EPCAM, and DAPI staining in PDO-CRC-87 following ANKRD54 or ELOVL6 knockdown (Scale bar = 50 μm). ROS (Green), EPCAM (Pink) and DAPI (Blue). Luminescence of ROS staining was calculated and compared from 10 random fields per sample (****P*<0.001, ns= no significance).

**J** Representative confocal images showing 4-HNE and DAPI staining in CRC-45 PDO following ANKRD54 or ELOVL6 knockdown (Scale bar = 50 μm). 4-HNE (Red) and DAPI (Blue). Luminescence of 4-HNE staining was calculated and compared from 10 random fields per sample (****P*<0.001).

**K** Representative images of CRC-45 PDO following ANKRD54 or ELOVL6 knockdown, stained with DAPI (blue) and C11-BODIPY (Red, reduced form of C11-BODIPY; Green, oxidized form of C11-BODIPY). Scale bar =50 μm. Oxidized/Reduced C11 Bodipy fluorescence was calculated and compared from 10 random fields per sample (***P*<0.01, ****P*<0.001).

**L, M** Western blot analysis of ACSL4, FTH1, FDFT1, HMOX1, HSPB1 and SLC7A11 in HT29, RKO and HCT116 cells treated with ANKRD54 (K) or ELOVL6 (L) siRNAs.

**N** Representative confocal images showing PTGS2, EPCAM, and DAPI staining in CRC-45 PDO transfected with shANKRD54 or shELOVL6 vector (Scale bar = 50 μm). PTGS2 (Red), EPCAM (Pink) and DAPI (Blue). Luminescence of PTGS2 staining was calculated and compared from 10 random fields per sample (****P*<0.001).

**O, P** Bright-field microscopy images of CRC-87 and CRC-45 PDOs (N) and organoid derived from WT and Vil-CRE;BRAF^LSL-V637E/+^ mice colorectal tissues (O) with ELOVL6 knockdown. Organoid diameters were measured using ImageJ (Scale bar = 100 μm, ****P<*0.001, ns= no significance).

Data information: For (A-H, O, P), statistical significance was determined by the two-tailed Student’s t-test. For (I-K, N), statistical significance was determined by the nonparametric Mann-Whitney test.

**FIG S7**

**A** Schematic of chemical structure of ELOVL6-IN-3.

**B, C** Determination of IC50 for ELOVL6-IN-3 in PDOs (B) and mouse organoids (C).

**D-F** qRT-PCR assays of ELOVL family members (ELOVL1, ELOVL2, ELOVL3, ELOVL4, ELOVL5, ELOVL6, and ELOVL7) mRNA levels in HT29 (D), RKO (E), and HCT116 (F) cells treated with ELOVL6-IN-3 at indicated concentration (biological replicates, n=3; **P<*0.05, ***P<*0.01, ****P<*0.001, ns= no significance).

**G** Western blot analysis of ELOVL5, ELOVL6, and ELOVL7 protein levels in HT29, RKO and HCT116 cells treated with ELOVL6-IN-3 at indicated concentration.

**H** Representative confocal images showing ELOVL6, Ki-67, EPCAM and DAPI staining in CRC-87 and CRC-45 PDO treated with ELOVL6-IN-3 at indicated concentration (Scale bar = 50 μm). ELOVL6 (Red), Ki-67 (Green), EPCAM (Pink) and DAPI (Blue). Luminescence of ELOVL6 staining was calculated and compared from 10 random fields per sample (****P<*0.001).

**I, J** Safety and tolerability assessment of ELOVL6-IN-3 *in vivo*, showing mouse body weight (I) and organ histology (J).

Data information: For (D-F, I), statistical significance was determined by the two-tailed Student’s t-test. For (H), statistical significance was determined by the nonparametric Mann-Whitney test.

**FIG S8**

**A** qRT-PCR determination of KAT2A, CEBPB, ELOVL6 and PTGS2 mRNA levels of ANKRD54-low expression and ANKRD54-high expression BRAF WT CRC patients in Cohort 1 (n=122; KAT2A: *P*=0.1422; CEBPB: *P*=0.9433; ELOVL6: *P=*0.3156; PTGS2: *P*=0.4358).

**B** Correlation analysis of ANKRD54, KAT2A, CEBPB, ELOVL6, and PTGS2 in BRAF WT CRC of Cohort 1 (n=122, ****P<*0.001, ns= no significance).

**C** Representative IHC images and statistical analysis of ANKRD54, KAT2A, CEBPB, ELOVL6, and PTGS2 in BRAF WT CRC tissues with higher or lower ANKRD54 expression samples from Cohort 2 (n=75). Right panels show enlarged images of indicated BRAF WT CRC tissues. Scale bars = 100 μm (left) and 10 μm (right).

**D, E** CERES dependency score for ANKRD54 across CCLE CRC cell lines with BRAF status (*P*=0.0363), TP53 status (*P*=0.9210), KRAS status (*P*=0.5232), NRAS status, APC status (*P*=0.8035), and PIK3CA status (*P*=0.1751).

**F** CERES dependency score for ANKRD54 across all CCLE cell lines with NRAS status (*P*=0.2399), APC status (*P*=0.2428), and PIK3CA status (*P*=0.7384).

Data information: For (A, D-F), statistical significance was determined by the two-tailed Student’s t-test. For (B), statistical significance was determined by analysis of the Pearson correlation coefficient. For (C), statistical significance was determined by the Fisher exact test.
